# Unfolding the Photophysical Behavior of Luminescent Polymeric Films Containing β‑Diketonate Tetrakis Eu^III^ Complexes via Multilayer Quantum Mechanics

**DOI:** 10.1021/acsomega.5c02434

**Published:** 2025-07-12

**Authors:** Leonardo F. Saraiva, Ariane C. F. Beltrame, Airton G. Bispo-Jr, Felipe S. M. Canisares, Albano N. Carneiro Neto, Renaldo T. Moura Jr, E. Kraka, Sergio A. M. Lima, Ana M. Pires

**Affiliations:** † Department of Chemistry and Biochemistry, School of Science and Technology, 28108 São Paulo State University (UNESP), 19060-900 Presidente Prudente, Brazil; ‡ Institute of Biosciences, Humanities and Exact Sciences, Department of Chemistry and Environmental Sciences, São Paulo State University (UNESP), 15054-000 São José do Rio Preto, Brazil; § Department of Physics, University of Aveiro, 3810-193 Aveiro, Portugal; ∥ Department of Sustainable Development and Ecological Transition (DISSTE), University of Eastern Piedmont “A. Avogadro”, 13100 Vercelli, Italy; ⊥ Institute of Chemistry, University of São Paulo (USP), 05508-900 São Paulo, Brazil; # Academic Unit of Cabo de Santo Agostinho, 67744 Federal Rural University of Pernambuco (UFRPE), 54518-430 Cabo de Santo Agostinho, Brazil; ∇ Department of Chemistry (Computational and Theoretical Chemistry Group), 2765 Southern Methodist University (SMU), 75725 Dallas, Texas, United States

## Abstract

The elucidation of the mechanisms governing the photophysical properties of trivalent lanthanide (Ln^III^) complexes embedded in polymeric matrices, such as poly­(methyl) methacrylate (PMMA) and polyvinylidene fluoride (PVDF), is challenging due to their intricate composite nature. Although theoretical modeling offers insights into film luminescence, conventional computational strategies face significant limitations. Given the large-scale nature of these systems, which encompass thousands of atoms, full-scale quantum mechanical (QM) simulations are impractical. Existing methodologies often integrate molecular mechanics (MM) with QM, yet such hybrid frameworks introduce inaccuracies in excited-state calculations. To address these challenges, this study proposes a multilevel computational protocol employing a hybrid QM/QM approach. Density functional theory (DFT) was used for the [Eu­(dbm)_4_]^−^ complex (dbm = 1,3-diphenyl-1,3-propanedione), while the polymeric environment was treated using the GFNn-xTB method. The protocol demonstrated strong agreement between experimental and theoretical emission quantum yields (QY) for both the isolated complex and [Eu­(dbm)_4_]^−^/PMMA or PVDF films. Our findings revealed that angular and length distortions in the Eu–O bonds within PVDF were induced by increased packing around the complex, impacting the ligand-Eu^III^ energy transfer by elevating the triplet state energy. These results underscore the predictive capabilities of the hybrid QM/QM strategy, offering a robust alternative for deciphering opto-structural relationships in Ln^III^-based polymeric films.

## Graphical abstract

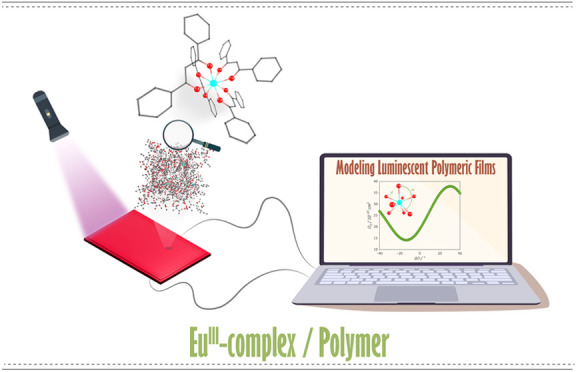

## Introduction

A powerful technique in materials science is to manipulate the structure and composition of materials to control their physical properties.^1^ This strategy is widely employed among chemists to optimize material properties for practical market applications. A prominent example is the enhancement of the optical response of molecular cluster-aggregates under ultraviolet (UV) excitation through composition control.^2,3^ This methodology can be easily extended to other systems, particularly those based on trivalent lanthanide ions (Ln^III^), which have been extensively studied due to their intriguing photophysical features. These characteristics include, but are not limited to, sharp emission bands spanning from the near-UV (NUV) to the near-infrared (NIR) spectral regions, high emission quantum yield (QY), and high emission color purity resulting from intraconfigurational 4*f*↔4*f* transitions.^4^ ,^5^ Due to the forbidden nature of these transitions in free ions, direct sensitization of Ln^III^ is inefficient. Consequently, emission-enhancing techniques, such as the coordination of Ln^III^ with organic chromophores are essential,^6^ as the ligand can act as an antenna, absorbing energy and transferring it to the Ln^III^ ion.

Among the wide range of organic chromophores, β-diketone ligands have emerged as desirable luminescent antennas,^7^ producing bright compounds with notable QYs because of their suitable energy levels for sensitizing Ln^III^.^8^ In this context, tetrakis complexes have been extensively studied in literature,^9,10^ as the introduction of a fourth ligand in the coordination sphere can enhance the emission QY by replacing coordinated water molecules and increasing the structural rigidity.^11^ Despite significant advances in the development of these complexes, a gap remains in transitioning from molecular designs to materials and ultimately to practical devices, primarily due to their low thermal and photostability.^12,13^ To address this challenge, a viable approach lies in dispersing the Ln^III^ complex into polymeric matrices to form luminescent films, thereby improving mechanical, thermal, and optical properties. This strategy entails more attractive materials for potential applications in light-emitting diodes (LEDs),^14^ organic-LEDs (OLEDs),^15^ luminescent solar concentrators (LSCs),^16^ and anticounterfeiting measures.^17,18^ Various polymeric matrices have been explored for this purpose, e.g., poly­(methyl) methacrylate (PMMA) and polyvinylidene fluoride (PVDF), due to their desirable mechanical and spectroscopic properties.^19–21^

Dispersing Ln^III^ complexes into polymeric matrices often leads to several changes in the photophysical characteristics of the emitter.^12,20,22^ These alterations may arise from complex-polymer interactions, which typically lead to structural distortions, modifying the rates of energy transfer (ET) from the antenna to the 4*f* center and directly affecting its sensitization. At first glance, classical spectroscopic techniques can provide valuable initial insights into the photophysical properties of luminescent films; however, they fall short in elucidating mechanisms and features at the atomic and subatomic scales.^23^ In such cases, theoretical and computational tools have emerged as viable solutions, offering detailed information and deeper understanding.^24,25^ This information is harnessed by modeling the Ln^III^-based complex, which requires a robust level of theory that accounts for relativistic effects to accurately describe the intricate nature of the 4*f* center.^26,27^ When a complex is embedded in a polymeric matrix, the entire system may consist of hundreds to thousands of atoms, making full-scale relativistic simulation infeasible.^28^ Therefore, only the coordination compound can be treated at this level of theory.

From this perspective, the treatment of large systems often relies on classical force-fields (FFs) such as *CHARMM* ^29,30^ or *Amber*.^31^ The primary advantage of FFs is their ability to replace costly and complicated electronic structures with classical energy expressions driven by chemical knowledge. However, the required modeling parameters are available for only a limited number of elements,^32^ which may restrict their applicability in interdisciplinary research. In recent years, self-parametrized semiempirical quantum mechanical (SP-SQM) methods, such as the tight-binding GFNn-xTB approach,^33^ have addressed the shortcomings of standard FFs while improving the accuracy of conventional SQM approaches.^33,34^ This advancement has significantly contributed to the field of computational materials by enabling the combination of SP-SQM protocols with higher-level methods, such as density functional theory (DFT). This integration spawns multilevel simulations (QM/QM), allowing large systems,^35^ such as luminescent films, to be modeled by treating the complex with DFT and the polymer with GFNn-xTB.^35^

In contrast to these simulations, modeling the photophysical properties of Ln^III^ follows a well-established framework based on Judd–Ofelt theory^36,37^ and the ligand-to-Ln^III^ intramolecular energy transfer (IET).^4,38–40^ Although these frameworks have been extensively applied to phosphors,^41^ glasses,^42^ and various coordination compounds,^43^ their adaptation to the photophysical properties of complex-polymer films is absent in the literature. This lack of information raises questions about the effects at the atomic and subatomic levels induced by dispersing the complex in the polymer, as well as the perturbation of the excited-state dynamics of these compounds. Therefore, providing models and conducting in-depth analyses of the luminescence-governing dynamics in Ln^III^-based polymeric films can advance our understanding of their photophysical properties.

Motivated by the pursuit of advancing such understanding, we take on this challenge by introducing a theoretical investigation into the photophysical properties of [Eu­(dbm)_4_]­Q/PMMA and [Eu­(dbm)_4_]­Q/PVDF luminescent films (dbm = 1,3-diphenyl-1,3-propanedione, Q = didodecyltimethylammonium, C_26_H_56_N^+^). The [Eu­(dbm)_4_]­Q complex was chosen due to its well-elucidated properties, which were previously reported by our research group in a comprehensive experimental study.^44^ Regarding polymers, PMMA and PVDF exhibit distinct crystallinity,^45^ introducing an intriguing factor for the investigation. By varying the concentration of the complex (0.25–2 wt %) dispersed in the polymer,^14^ the photophysical properties changed significantly, underscoring the importance of studying both systems. Beyond elucidating these aspects, we established a correlation between the emission QY and structural distortions induced by switching the polymer from PMMA to PVDF. The outcomes obtained reinforce the feasibility of our proposed protocol, and investigating these mechanisms further has the potential to contribute to the development of Eu^III^-complex/polymer films with enhanced photophysical features.

## Methodology

### Isolated Complex

The initial geometry optimization was performed using a tight grid in the Orca 5.0.3 package^46^ at the DFT level, employing the r^2^SCAN-3c functional. This functional is known for its highly accurate description of large structures^47^ and intrinsically incorporates the D4 dispersion correction.^48^ All atoms were described by the all-electron Def2-TZVP basis set,^49^ except for Eu^III^, which was treated with “large-core” MWB52 effective core potential (ECP) alongside its corresponding valence basis set.^50,51^ After optimization, the minimum energy structure was subjected to a frequency calculation using the numerical Hessian method in the same software with the ωB97x-D4 functional^52^ due to its similarity to r^2^SCAN-3c while offering enhanced accuracy in excited-state energies calculations. This step was necessary to ensure the absence of imaginary frequencies.

### Complex/Polymer Film

A miniature chain comprising six monomer units of PMMA and PVDF in the absence of the complex was generated using Packmol software.^53^ The miniature chains of both polymers underwent geometry optimization using the r^2^SCAN-3c functional with the Def2-TZVP basis set.^48,49^ Subsequently, the complex/polymer film was constructed by spawning 200 interconnected-miniature chains around the complex. This system was then subjected to geometry optimization based on a self-parametrized force-field calculation using the GFNn-ff method. Starting from the minimum energy configuration of the system, multilevel QM/QM time-dependent DFT (TD-DFT) calculations were performed. In this calculation, the complex was treated using the ωB97x-D4/MWB52/Def2-TZVP method, whereas the polymer was treated with GFN2-xTB. The main aim of this calculation was to extract the energies of the S_1_ and T_1_ states along with the necessary parameters to determine the IET rates of the complex under polymer influence. It should be highlighted that the counterion was not considered in the calculation, as it could lead to complications due to the large number of atoms that should be treated at high levels. Concurrently, a conformer search was conducted using CREST software^54^ to obtain optimized conformers with energies similar to those of relaxed complexes. The overall procedure is depicted in Figure 1.

**Figure 1:**
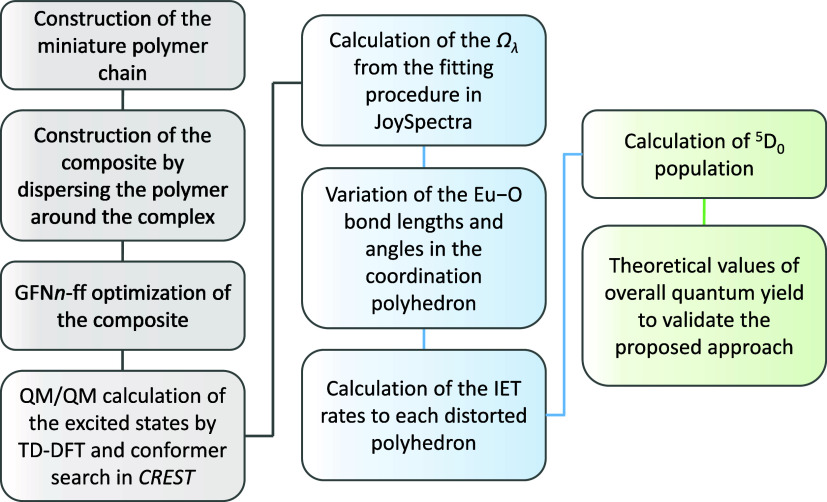
Flowchart for calculating the photophysical parameters for [Eu­(dbm)_4_]^−^ in PMMA or PVDF films. The colors in the boxes refer to each step; gray = structural simulations, excited states, and vibrational frequency calculations; blue = determination of Judd–Ofelt intensity parameters (Ω_λ_) and IET varying the distortion in the coordination polyhedron; green = computation of the population of the emitter level and theoretical overall emission quantum yield for each film.

### Theoretical Intensity Parameters

The theoretical Judd–Ofelt intensity parameters play a crucial role in providing insights into the chemical environment surrounding Eu^III^.^55^ These parameters include the charge factor (*g*_*j*_) and the effective polarizability (α′). The former was calculated as outlined in reference^56^ using local mode force constants determined from our local vibrational theory,^57^ while the latter was obtained through a fitting procedure detailed elsewhere.^58^ The experimental parameters varied with concentration; therefore, we adjusted the angles and lengths of the Eu–O bonds in the Eu^III^ coordination polyhedron in *JOYSpectra* ^59^ to match the experimental values. These experimental values were obtained from a previous study.^14^ The geometries resulting from these distortions were then used for intramolecular energy transfer modeling. The equations employed in this step are summarized in the Supporting note S2.

It should be emphasized that, for this step, photophysical calculations were performed only on the optimized structure, as the simulation of intensity parameters depends solely on the coordination polyhedron. Consequently, a systematic evaluation of the photophysical properties for each possible conformer identified in the previous step was deemed unnecessary.

### Intramolecular Energy Transfer

The rates of intramolecular energy transfer (IET) from the antenna ligand to Eu^III^ were computed by considering three mechanisms: dipole–dipole (*W*_d–d_), dipole–multipole (*W*_d–m_), and exchange mechanism (*W*_ex_). These calculations were performed according to eqs S8–S16.^60^ In all equations, *R*_L_ represents the donor–acceptor distance, which was determined using TD-DFT calculations. This parameter is crucial for estimating the rates because *W*_d–d_ depends on the sixth power on *R*_L_, *W*_m–d_ depends on the λ*+2* on *R*_L_, and *W*_ex_ on the fourth power of the donor–acceptor distance. The IET rates were calculated using the equivalent geometry of each distortion, which is necessary to harness the experimental values of the Judd–Ofelt parameters.

### Rate Equations and Overall QY

After computing the IET rates, a system of coupled ordinary differential equations (ODEs) was formulated and solved numerically using time propagation to determine the populations of each step in the IET. The set of ODEs was solved using the Radau method, which has been successfully employed in previous studies and has provided reliable results at a feasible computational cost.^61^ Thus, each level can be described by a general formula (eq 1):

1 $$\frac{dP_{i}\left(t\right)}{dt}=\sum_{j=1}W_{j\to i}P_{j}\left(t\right)-\sum_{j=1}W_{i\to j}P_{i}\left(t\right),i\neq j$$

where *P*_*i*_(*t*) and *P*_*j*_(*t*) represent the populations of the *i*- and *j*-level at time *t*, respectively. *W*_*j*→*i*_ denotes the rate of energy transfer from level *j* to level *i*, while *W*_*i*→*j*_ is the corresponding backward pathway (from *i* to *j*).

Each simulation was conducted over a time interval ranging from 0–10 ms with a fixed step-size of 1 μs. By harvesting the population of the emitting level of Eu^III^ (^5^D_0_) and the ground state (S_0_) after excitation, the theoretical overall quantum yield can be obtained from eq 2:

2 $$\Phi_{L}^{\mathrm{Eu}}=\frac{\mathrm{number}\;\mathrm{of}\;\mathrm{photons}\;\mathrm{emitted}}{\mathrm{number}\;\mathrm{of}\;\mathrm{photons}\;\mathrm{absorbed}}=\frac{A_{\mathrm{rad}}P_{E}}{\phi P_{0}}$$

where *A*_rad_ is the spontaneous emission coefficient calculated from the Judd–Ofelt intensity parameters,^62^ *P*_E_ represents the population of the emissive state, *P*_0_ the population of the initial state after excitation, and ϕ denotes the pumping rate. ϕ is directly determined by the expression ϕ = σρλ_exc_/*hc*,^63^ where σ is the absorption cross-section (value of ∼10^–16^ cm^2^ molecule^–1^), λ_exc_ is the excitation wavelength, *h* is the constant of Planck, *c* is the speed of light, and ρ is the power density, which is assumed to be 1 W cm^–2^ in our simulations.

## Results and Discussion

### Investigation of Film Structure

The electronic structure of an Ln^III^ coordination compound is intrinsically connected to its symmetry;^64^ therefore, accurate geometries are essential when modeling its photophysical properties. This dependence implies that even slight distortions in the ligand scaffold or the Eu^III^ microssymmetry can lead to fluctuations in the photophysical features of Eu^III^-based complexes.^55^ Accordingly, the complex dispersed in PMMA and PVDF was immobilized in the ground state, revealing that the structures belong to a C_1_ point group (Figure 2a,b) in both environments. A noticeable degree of distortion was observed between the optimized structures compared with the complex without the polymer. This distortion was qualitatively assessed using the root-mean-square deviation (RMSD), with a value of 0.17 for PMMA and 0.26 for PVDF. One contributing factor to this distortion is the shortening of Eu–O bond (Table S2) in the PVDF environment compared with PMMA, which can be attributed to the packing between the polymer moieties. As evidenced by the polymer structure (Figure S1), PMMA monomers are bulkier than PVDF because of the ester groups attached to the quaternary carbon, resulting in a more spaced distribution around the complex compared to PVDF (Figures S2–S3). Within this framework, since [Eu­(dbm)_4_]^−^/PVDF exhibits denser packing than [Eu­(dbm)_4_]^−^/PMMA, shorter Eu–O bonds are observed for the complex in the PVDF environment (Figure S4).

**Figure 2:**
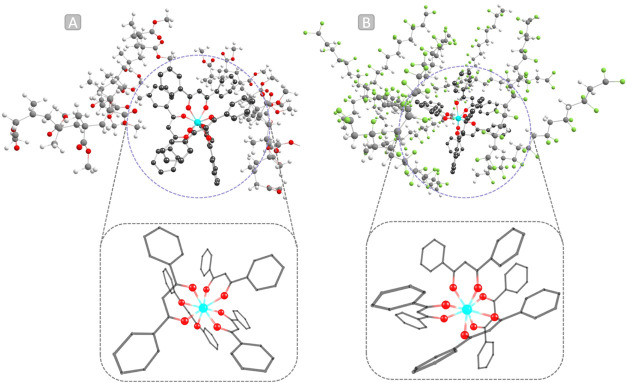
Ground-state QM/QM optimizations of [Eu­(dbm)_4_]^−^ in (a) PMMA and (b) PVDF, taken from Figure S3. The complex was treated at the high-level (DFT) and the polymer at the low-level (GFN2-xTB). Hydrogen atoms were hidden for clarity. The color code is as follows: cyan = europium, gray = carbon, green = fluorine, and red = oxygen.

Another important aspect to consider is the electronegativity of the constituent atoms of the polymer moieties. As the polymer and the complex come in closer proximity, the oxygen atoms in PMMA or the fluorine atoms in PVDF can interact with hydrogen from the ligand scaffold of the complex, leading to tighter packing.^65^ This aligns with the expected electronegativity trend, as F···H interactions are stronger than O···H due to fluorine’s higher electronegativity. However, relying solely on packing may not fully address the observed photophysical features described in the next section. Another perspective was obtained by comparing the [Eu­(dbm)_4_]^−^ structure in PMMA and PVDF with the isolated complex. Geometry optimization revealed that the aromatic ring of the isolated complex can rotate out-of-plane (Figure S3). However, when embedded in PMMA, this rotation is practically restricted by short- and long-range interactions between the complex and the polymer’s organic groups, allowing only minor rotations due to the available space within the packing. Despite this effect, the restriction was even more pronounced in PVDF, which possesses a semicrystalline^45^ and less bulky structure.^66^

It is noteworthy that the computational model employed presupposes an isotropic dispersion of complex particles throughout the polymer matrix. Elemental maps acquired by energy-dispersive X-ray spectroscopy (EDS, Figures S5–S8) demonstrate Eu^III^ agglomerates dispersed throughout the polymer – a typical behavior of complex/polymer films.^67–69^ Yet, PVDF comprises coexisting amorphous and crystalline phases, and given the resolution limits of the equipment, it is not possible to guarantee equivalent interactions with both structural regimes.^70^ Consequently, the simulation treats the complex as residing in an amorphous environment, omitting periodic boundary conditions. Incorporating these conditions would have increased the computational cost, which is beyond the scope of the proposed protocol. Furthermore, a full structural characterization of these films was provided in our earlier work.^14^

A wealth of information can be obtained from the analysis of the optimized geometries (Figure 2), particularly concerning the coordination polyhedron in Figure S4, which exhibits a distorted square antiprismatic geometry due to the 8-fold coordination. However, the interpretation of these findings requires caution because variations in symmetry can be implicated depending on the influence of the polymer during film production. An exploration of the conformers revealed the existence of three additional analogous structures in the presence of PMMA and PVDF (Figure 3) with energies similar to those of the optimized system at the QM/QM level. Among these geometries, conformers number three in PMMA and number two in PVDF exhibit *C*_s_ symmetry in the coordination polyhedra. Although these groups represent low-symmetry point groups, this alteration confirms that structural distortions are often influenced by packing effects. Moreover, this outcome suggests that multiple conformations may be present in the film. However, this aspect is beyond the scope of this work because it introduces complexities that cannot be addressed using the software and approximations employed.

**Figure 3:**
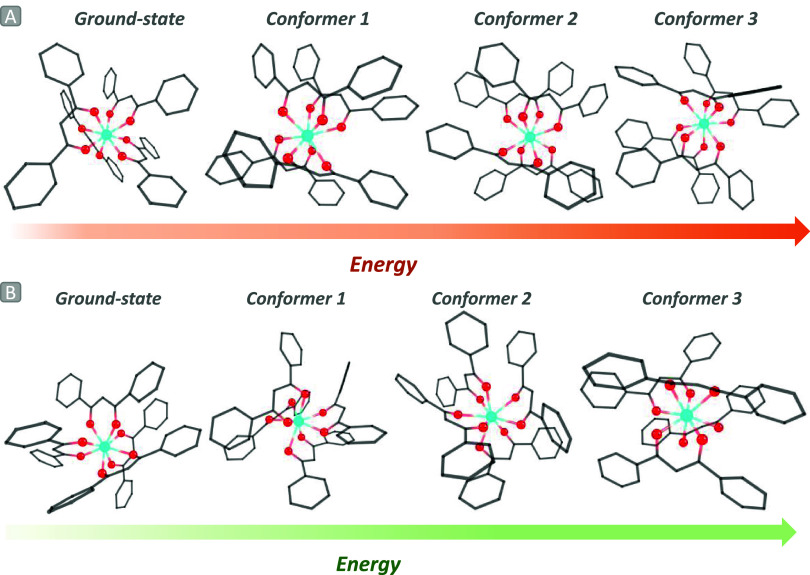
Optimized ground-state at the QM/QM level and their respective conformers in (a) PMMA and (b) PVDF using *CREST* software

### Theoretical Judd–Ofelt Analysis

With an atomic-level representation of the molecular distortion in both environments of the complex, it becomes feasible to delve into the subatomic scale of these compounds, particularly their electronic structure. The emission bands assigned to intraconfigurational 4*f*↔4*f* transitions are explained by the Judd–Ofelt theory, whose centerpiece lies in three intensity parameters (Ω_2,4,6_), from which the transition probabilities, radiative lifetime, and other photophysical features can be directly obtained.^71,72^ For Eu^III^-based compounds, these parameters are directly associated with the emission bands assigned to ^5^D_0_→^7^F_2,4,6_ electronic transitions, and they vary according to their rates, using the magnetic dipole allowed ^5^D_0_→^7^F_1_ transition as reference.^26^ However, Ω_λ_ (λ = 2, 4, 6) are often interpreted beyond their phenomenological nature, being directly correlated with chemical trends, such as distortions, ligand polarizability, rigidity, covalency, among others.^73^

From this perspective, Ω_2_ is widely used throughout papers to describe the degree of distortion in Eu^III^ microssymmetry in terms of Eu–L angular variations,^55,74^ while Ω_4_ is said to reflect changes in the Eu–L bond covalency.^41,55^ Although these qualitative aspects have been discussed for Eu^III^-based complexes,^55^ it is important to question whether quantitative trends align with qualitative ones. The answer to this question lies in Figure 4, where we varied the spherical coordinates (*r,θ,φ*) of a certain plane of atoms in the Eu^III^ coordination polyhedron (Figures S9–S10). It is worth emphasizing that any influence of alternative conformers can be effectively accounted for by adjusting the coordinates of the ligand atoms within the coordination polyhedron. The behavior of the Ω_6_ parameter with these distortions will not be studied because the ^5^D_0_→^7^F_6_ emission was not observed due to the limitations of the apparatus.^14^

**Figure 4:**
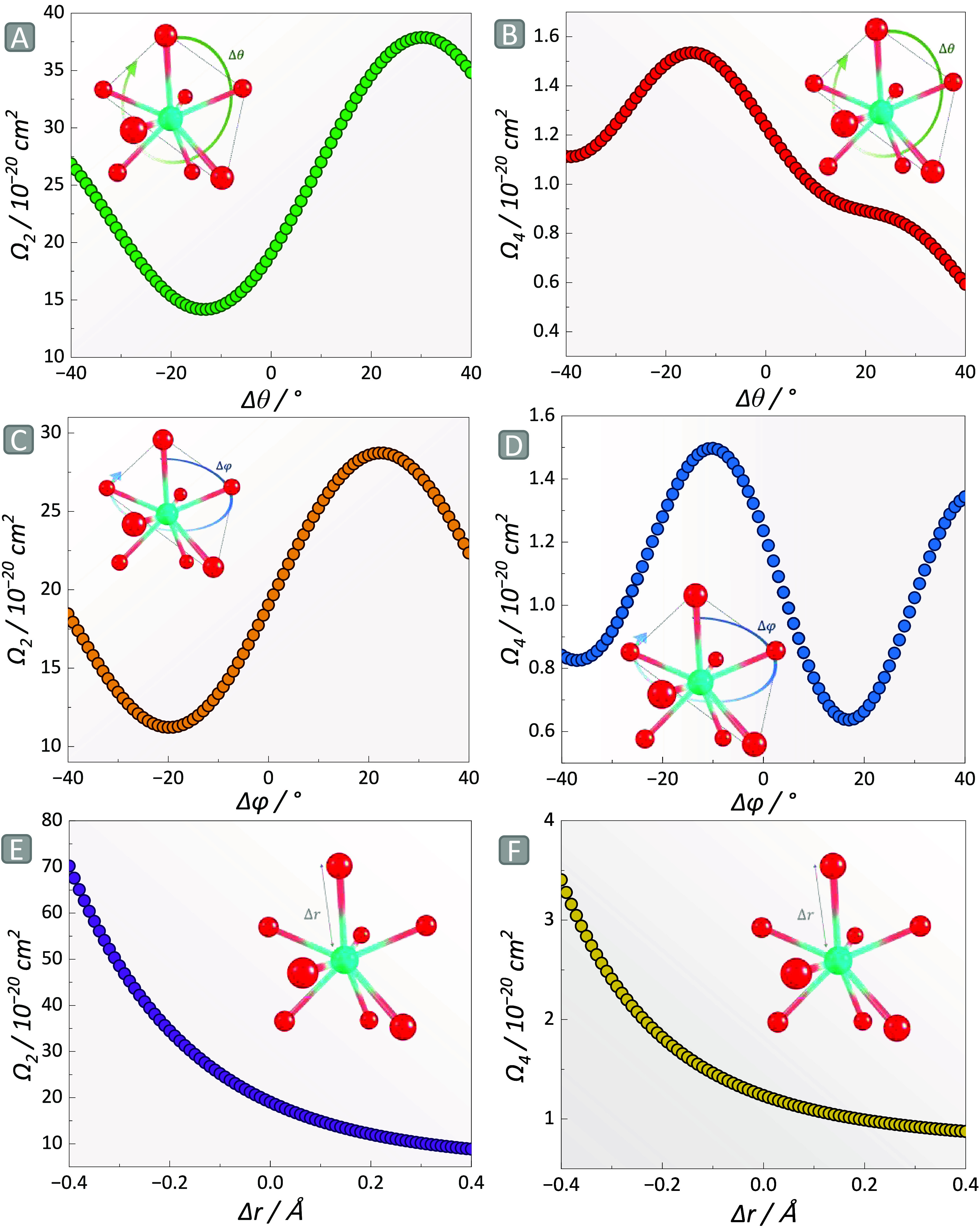
Trends in the theoretical Judd–Ofelt intensity parameters in [Eu­(dbm)_4_]^−^/PMMA by varying the θ angle in (a) Ω_2_ and (b) Ω_4_, φ angle in (c) Ω_2_ and (d) Ω_4_, followed by variations in the Eu–O distances in (e) Ω_2_ and (f) Ω_4_.

The variation of Ω_2_ in terms of θ and φ followed a sinusoidal form by varying 1.5° to an adaptive step in the – 40 to 40° range, where the absolute values derived from Figure 4 are presented in Tables S3–S8. This change can be correlated with those promoted by altering the concentration of the complex in PMMA, which modified the experimental parameters due to the chemical environment around [Eu­(dbm)_4_]^−^. By considering the angles θ and φ, the plane of oxygen is distorted in the – 11 to 12° range, where a negative degree indicates a deformation in the counterclockwise direction. An interesting outcome of this analysis is that not every angular deformation augments the value of Ω_2_, as the sinusoidal shape tends to decrease after its peak in the 20–25° region in the clockwise direction. These observations stem from the induction of a more symmetric environment at higher angle distortions, which becomes less symmetric when the sinusoidal shape is repeated. This hypothesis is experimentally interpreted as the variation in the emission band associated with ^5^D_0_→^7^F_2_ transition, simultaneous to the induced distortion.

Concomitantly with θ and φ, varying the Eu–O bond lengths yielded the trend shown in Figure 4e, where an exponential dependence of Ω_2_ with Δ*r* was noticed. This behavior is driven by the hypersensitive character of ^5^D_0_→^7^F_2_ transition, explained by the dynamic coupling (DC) mechanism,^75^ which lies in the polarizability of the chemical environment surrounding the Ln^III^.^76^ In this context, Ω_2_ reflects both the angular distortion and polarizability of Eu^III^ microssymetry. However, the dominant effect remains elusive since both phenomena occur simultaneously. Thus, using Ω_2_ to probe properties such as covalency may not always yield the correct outcome.

Regarding the Ω_4_ parameter, it becomes evident from Figure 4b the nondefined shape of the variations in the values by distorting the θ angles, peaking in the counterclockwise 20° region. In contrast, changing φ yielded a well-defined sinusoidal pattern with a maximum value of 1.52 × 10^–20^ cm^2^. The lower dependence of Ω_4_ on the angular distortions is noteworthy in this analysis. To understand this behavior, the phenomena can be analyzed from both physical and chemical perspectives. From a physical standpoint, when the Eu^III^ coordination polyhedron becomes more symmetrical, the odd-crystal field quantities, particularly Γ_p_^t^ (representing the DC mechanism), with lower ranks tend to approach zero more rapidly than those with higher ranks, as they are more dependent on the spherical coordinates.^55,76^ In this context, lower-rank quantities are more sensitive to angular distortions, meaning that Ω_2_ is more responsive to θ and φ than Ω_4_ and, subsequently, Ω_6_. From a chemical perspective, the nearly symmetry-independent character of ^5^D_0_→^7^F_4_ transition results in Ω_4_ only slightly affected by angular deformations.^23,74^ Analogous to the exponential trend of Ω_2_ with fluctuations in bond length, a similar pattern is observed for Ω_4_ (Figure 4f), where this parameter is highly sensitive to these displacements. In this case, it is worth noting that the negative values of Δ*r* express lengths shorter than the equilibrium bond length. Another important aspect that can be derived is that the participation of bond overlap polarizability (Table S9), a measure of the covalency degree,^77^ is higher for Ω_4_ (Table S10), suggesting that Ω_4_ is more suited to investigate the covalency trends for this complex.

The same analysis can be extended to the [Eu­(dbm)]^−^/PVDF film, where the trends in the Judd–Ofelt intensity parameters are highlighted in Figure S11. All variations followed a pattern similar to that of the PMMA film, except for the changes in Ω_2,4_ when varying θ. The values of Ω_2_ are higher for PVDF than for PMMA due to larger distortions and the induction of a more polarizable environment, which was observed due to the greater packing, as previously discussed. As the PVDF molecules approach the complex, the complex undergoes distortions that amplifies its polarizability. Therefore, a combination of these two factors enhances the Ω_2_ by 2-fold compared with the isolated complex. To achieve the correct theoretical values, distortion occurred in the plane of oxygen in the equatorial region instead of the axial region because the oxygen in the out-of-plane region of Figures S9–S10 forms an almost symmetrical plane. Thus, when this Eu–O bond is deformed, this possibility is restricted, leading to higher Ω_2_. From a chemical perspective, we can associate these values with the rates of ^5^D_0_→^7^F_2_ transition, because in the Wybourne formalism, a higher number of odd terms (28) are found for C_1_ symmetry than for *C*_s_ (16 terms),^78,79^ elevating the intensity of the band.

Despite the variations in Ω_2_, the most pronounced difference between PMMA and PVDF films in terms of Judd–Ofelt analysis lies in the 6-fold increase of Ω_4_ (Tables S11–S16). An interesting outcome provided by Figure S11 is that no angular variation (θ,φ) resulted in the experimental values of Ω_4_, implying that deformations in Eu^III^ microssymetry play a minor role in these values. However, when varying the Eu–O bond lengths, the theoretical values approached the experimental ones, decreasing the lengths to an extent of 0.5 Å, which is higher than all displacements observed for the PMMA film.

As previously demonstrated, parameter Ω_4_ is better suited to evaluate covalency trends because of the higher contribution of bond overlap polarizability (α_OP_). In this sense, Figure S12 displays the overlap integral and the α_OP_ in terms of absolute variation in bond length (*R*′ = *R*_eq_ + Δ*r*, where *R*_eq_ represents the equilibrium length, Δ*r* the variations in Figures 3 and 4, and *R*′ the absolute variation). Because the equilibrium bond length was taken as 2.529 Å, the variations reported in Table S16 were responsible for the experimental values of Ω_4_, leading *R*′ to nearly 2 Å, resulting in a 2.5-fold increase in the polarizability of the bond (Figure S12) and therefore the covalency degree. Hence, it is reasonable to suggest that in the PVDF film, the polymer molecules, along with their packing, induce a more distorted and covalent environment than PMMA, directly influencing other photophysical properties, such as the quantum yield of the film.

### Energy Transfer and Quantum Yield

The theoretical Judd–Ofelt intensity parameters enabled the exploration of various photophysical features of the complex within both polymeric environments. Among these properties, energy transfer (ET) assumes paramount importance because of its direct correlation with the emission QY.^62^ To determine ET rates, calculations were conducted via TD-DFT at the QM/QM level for both polymers to extract singlet and triplet energy positions alongside donor–acceptor distances. The values acquired are summarized in Tables S17–S19, accompanied by a graphical representation of the state composition in terms of molecular orbital (MO) contributions in Figures S13 and S14, which correspond to the complex in PMMA and PVDF, respectively.

A meticulous inspection of the isosurfaces of these MOs revealed an antibonding character concentrated within the high-electron-density region, i.e., the resonant region of the ligand scaffold. Notably, as these isosurface shift toward the aromatic rings, an increase in the donor–acceptor distance (*R*_L_) becomes apparent. *R*_L_, defined as the distance between the centroid position of the MOs weighted by their coefficients (donor) and Eu^III^ (acceptor), demonstrates that even small changes significantly affect intramolecular energy transfer (IET) rates.^60,61^ This sensitivity arises because of the dependence on the sixth power of the dipole–dipole and the fourth power of the ET exchange mechanism, as detailed in eqs S8–S10.

The rates of energy transfer and back-energy transfer (BET), derived by employing the values reported in Table S19, indicate the suitability of the ligand set for sensitizing the Eu^III^ ion, as the ET rate outperforms the BET (Tables S20–S21). This mitigates an additional deactivation pathway that could quench Eu^III^ emission. Intriguingly, although the *R*_L_ value of the complex in PVDF is lower, the ET of the T_1_ state (*W*_*T*_) for [Eu­(dbm)_4_]^−^/PMMA exceeds [Eu­(dbm)_4_]^−^/PVDF by an order of magnitude. This discrepancy arises from the lower energy of the T_1_ state of [Eu­(dbm)_4_]^−^/PMMA by 1000 cm^–1^, overshadowing the 0.02 Å difference in *R*_L_. Consequently, this difference leads to nearly a 2-fold increase in BET rates for [Eu­(dbm)_4_]^−^/PVDF compared to [Eu­(dbm)_4_]^−^/PMMA, quenching the luminescence by deactivating Eu^III^ excited levels.^80^ The increase in the BET is associated with reducing the energy gap in the primary BET pathway, i.e., T_1_←(^7^F_1_→^5^G_2_), which displays values of – 4229 and – 3230 cm^–1^ for the complex in PMMA and PVDF, respectively (Tables S20 – S21). Regarding ET stemming from the S_1_ level, a parallel trend is observed, where, for [Eu­(dbm)_4_]^−^/PMMA, the ET rates exceed those of [Eu­(dbm)_4_]^−^/PVDF due to the lower *R*_L_ values for S_1_ (Table S19). Therefore, Tables S20–S21 underscore that ET via the exchange mechanism predominates for the T_1_ state, where ^5^D_1_ is the main acceptor (considering the ^7^F_0_→^5^D_1_ pathway). Such a result means that ^5^D_0_ is mostly populated due to the relaxation of the ^5^D_1_ manifold. Concurrently, ET from the S_1_ state is primarily constituted by dipole and multipole interactions. However, high ET rates are obtained for the S_1_→(^7^F_1_→^5^G_2_) pathway, where the exchange mechanism emerges as the leading one. This phenomenon is rooted in ET selection rules for 2^λ^-pole, which entail |*J* – *J*′| ≤ λ ≤ *J* + *J*′, and for exchange, |*J* – *J*′| = 0, 1, thereby inhibiting the dipole and multipole contribution for the ^7^F_1_→^5^G_2_ pathway.^4,81^

Distorting the Eu^III^ coordination polyhedron varies the values of Ω_2,4_ as shown in the previous section, thereby altering the ET and BET rates. Given that the primary pathway of ET stems from the T_1_ state, our focus centers on this level to assess the IET rates by manipulating the spherical coordinates (*r*,θ,φ), as shown in Figure S15. Within this framework, we observe a nonlinear relationship between ET and coordinate variation for the complex in PMMA and PVDF. Despite this trend, the 2% film exhibited the highest ET value, implying that the sensitization of Eu^III^ reaches its peak at this concentration. Concurrently, the BET rates followed a similar pattern, with an exception observed in the case of 2 wt % [Eu­(dbm)_4_]^−^ in PVDF, where BET reached its lowest value. Interestingly, for PVDF, the rates of ET and BET remained the same until 1 wt % varying θ and φ angles, with the absolute values highlighted in Tables S22–S25. This outcome aligns with expectations given that Ω_2,4_ underwent identical variations in both scenarios.

To understand the origin of the photophysical characteristics of the films, exploring beyond ET rates becomes pivotal, considering other potential deactivation routes. To address this issue, a diagram depicting the ligand-to-Eu^III^ energy transfer pathways along with plausible deactivation mechanisms was proposed (Figure 5a). The above analysis led to the formulation of a set of coupled ODEs based on eq 1 outlined in the methodology, enabling the capture of population kinetics (eq 3). Within this set of ODEs, various states, such as the ground state (S_0_ at *t* = 0, transitioning to ^7^F_J_ at *t* ≠ 0), T_1_, and S_1_ are defined as |0⟩, |1⟩ and |2⟩, respectively. |3⟩ represents any Eu^III^ manifold other than ^5^D_0_, while |4⟩ is the ^5^D_0_ level. The lifetime decay values of S_1_, T_1_, and ^5^D_0_ states, assigned as τ_S_, τ_T_, and τ, respectively, assume values in the range of 1 ns–1 μs for τ_S_, 1 μs–1 ms for τ_T_, and 1 ms for τ.^82^ The intersystem crossing (ISC) rates denoted as *W*_ISC_ were estimated to be around 10^8^ s^–1^, considering the energy gaps between S_1_ and T_1_ within 10,000 to 15,000 cm^–1^,^61^ consistent with the studied complex (12457 cm^–1^ for [Eu­(dbm)_4_]^−^/PMMA and 11421 cm^–1^ for [Eu­(dbm)_4_]^−^/PVDF). The deactivation pathway: ^5^D_1_→^5^D_0_ (*W*_3→4_) ranges from 10^4^–10^5^ s^–1^, as presented in a previous study.^83^ It is worth noting that the population dynamics are heavily reliant on the chosen boundary conditions,^84^ ensuring that the total population across all energy levels remains constant at any given time *t* within the employed interval. The main outcome of this simulation is depicted in Figure 5b, highlighting a notably higher population of the ^5^D_0_ state for [Eu­(dbm)_4_]^−^/PMMA compared to [Eu­(dbm)_4_]^−^/PVDF, which aligns with the higher experimental quantum yield of the film reported in reference.^14^

3a $$|0\rangle :\frac{d}{dt}P_{0}=-\phi P_{0}+\frac{1}{\tau_{T}}P_{1}+\frac{1}{\tau_{S}}P_{2}+\frac{1}{\tau }P_{4}$$

3b $$|1\rangle :\frac{d}{dt}P_{1}=-\left(\frac{1}{\tau_{T}}+W^{T}+W^{T\prime }\right)P_{1}+W_{\mathrm{ISC}}P_{2}+W_{b}^{T}P_{3}$$

3c $$|2\rangle :\frac{d}{dt}P_{2}=-\left(\frac{1}{\tau_{S}}+W^{S}+W_{\mathrm{ISC}}\right)P_{2}+W_{b}^{S}P_{3}+\phi P_{0}$$

3d $$|3\rangle :\frac{d}{dt}P_{3}=-\left(W_{b}^{S}+W_{b}^{T}+W_{3\to 4}\right)P_{3}+W^{T}P_{1}+W^{S}P_{2}$$

3e $$|4\rangle :\frac{d}{dt}P_{4}=-\left(\frac{1}{\tau }\right)P_{4}+W^{T\prime }P_{1}+W_{3\to 4}P_{3}$$

**Figure 5:**
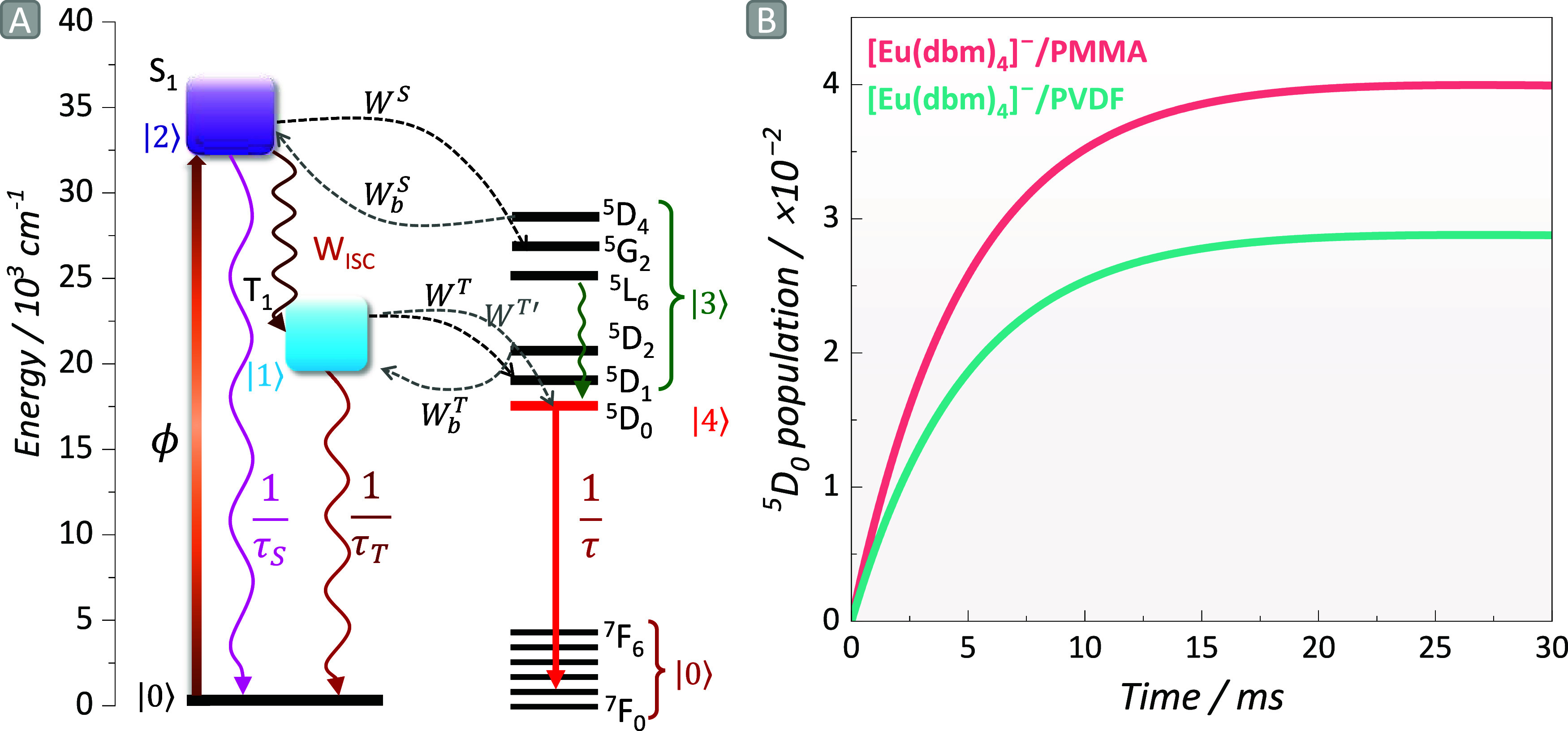
(a) Representative partial energy level diagram of the complex showing the ligand-centered singlet (S) and triplet (T) state energies as well as ^2S+1^L_J_ levels of Eu^III^. The ligand absorption (light orange arrow), intersystem crossing (ISC, intense orange), ligand-to-Eu^III^ energy transfer (ET, black and gray dashed arrows), back-ET (light gray dashed arrows), relaxation (green wavy arrow), and Eu^III^ emission (red arrow) processes are also represented. (b) Population kinetics of Eu^III 5^D_0_ excited state of the complex for the 0.50 wt % film.

From Figure S16,S17, notably, for PMMA, the most significant change in population with increasing complex concentration occurred by varying θ. The 2 wt % concentration demonstrated the highest population (0.0415) compared with the 0.25 wt % (0.0373), which is consistent with the highest experimental emission lifetime observed for this film, as indicated in reference.^14^ When varying φ and *r*, minimal population fluctuations were observed (Figure S17a), consistent with the energy transfer trends shown in Figure S15, where the rates reached their peak with θ (1.170 × 10^8^ s^–1^ for the T_1_). In contrast, PVDF exhibited nearly constant population behavior as a function of concentration, owing to the small alterations in ET rates with increasing doping amount. Consequently, population variations hovered around the 4 × 10^–4^ range (Figure S17b). This phenomenon underscores that the population alone may not be fully entitled to elucidate the emission quantum yield of films because the theoretical quantum yield values also rely on the radiative decay of ^5^D_0_, as outlined in the methodology.

Given the population values in the dynamic regime (Table S26), the pumping rate, and the theoretical radiative decay estimated from eqs S20–S22, we derive the theoretical values of quantum yield (**Φ**_**L**_^**Eu**^) summarized in Table 1, along with the aforementioned photophysical properties. Notably, in downshifting systems such as the ones under study, **Φ**_**L**_^**Eu**^ remains independent of the excitation power density.^16^ This observation stems from the fact that when the pumping rate is increased *n*-fold, the ratio between the population of the emitting level and the ground state is also enhanced by *n*-fold.^85^ This is due to the proportional augmentation of the emitting-level population alongside the depletion of the ground-state.

**Table 1:** Experimental and Theoretical Photophysical Parameters: Radiative Decay (*A*_rad_) and Overall Quantum Yield (Φ_L_^Eu^) of the Isolated Complex, PMMA, and PVDF Films^a^

**wt %**	***A***_**rad**_^**exp**^ **/s** ^**–1**^	***A***_**rad**_^**theo**^ **/s** ^**–1**^	**exp**. **Φ**_**L**_^**Eu**^**/%**	**theo**. **Φ**_**L**_^**Eu**^**/%**
	**[Eu(dbm)**_**4**_ **]** ^**–**^			
	652	663	6.40	6.98

	**[Eu(dbm)**_**4**_ **]** ^**–**^ **/PMMA**			
**0**.**25**	574	579	0.20	0.29
**0**.**50**	521	530	0.40	0.52
**0**.**75**	764	772	2.20	2.43
**1**.**00**	815	821	22.0	24.1
**2**.**00**	835	844	40.0	45.2

	**[Eu(dbm)**_**4**_ **]** ^**–**^ **/PVDF**			
**0**.**50**	944	951	2.70	2.95
**0**.**75**	970	979	14.0	16.2
**1**.**00**	879	886	5.80	6.21
**2**.**00**	1011	1025	21.0	23.3

a The pumping rate used to estimate the overall quantum yield was 198.9 s^–1^ under 395 nm excitation. All experimental values were adapted from the Supporting Information (Table S2) of reference 14, with permission from the Royal Society of Chemistry.

The reported values in Table 1 display a well-desired agreement between the theoretical and experimental values of the radiative decays and emission quantum yields. Interestingly, dispersing the complex within the polymer matrix led to a minimum 3-fold increase in the quantum yield at the highest complex concentration. This enhancement is associated with amplified distortions in Eu^III^ microssymmetry, since the trends in Ω_2_ indicate that as the concentration increases, angular deformations in the coordination polyhedron intensify. This potentially relaxes the constraints imposed by the selection rules for the ^5^D_0_→^7^F_2_ transition. This hypothesis is supported by experimental and theoretical trends in radiative decay rates. Another factor that needs to be considered is the escalated ET values from the triplet state with increasing concentration, which play a crucial role in the ^5^D_0_ population and influence the quantum yield.

From an experimental perspective, the ^5^D_0_ level is susceptible to deactivation by high-energy oscillations like O–H and O–O oscillators in water and air, respectively.^12,14,86^ Consequently, the polymer might play a protective role by avoiding a reduction in the radiative decay rate, thereby enhancing the emission quantum yield due to higher values of *A*_*rad*_. In contrast to [Eu­(dbm)_4_]^−^/PMMA, no clear trend was observed in the emission quantum yield for [Eu­(dbm)_4_]^−^/PVDF. Intriguingly, the 1 wt % PVDF sample exhibited a higher value of **Φ**_**L**_^**Eu**^ than the 0.50 wt % sample, despite having lower radiative decay (Table 1). However, in comparison to the 0.75 wt % sample, an anomalous trend is observed, since the latter presents a higher **Φ**_**L**_^**Eu**^. This counterintuitive occurrence can be explained by two main hypotheses: (*i*) faster ET due to the shortening of Eu–O bond induced by the PVDF. (*ii*) Faster depletion of the excited-state (Figures S16–S17), which reduces *P*_0_ that presents an inversely proportional relationship with the quantum yield (eq 2), resulting in higher values of **Φ**_**L**_^**Eu**^ compared to the 0.5 wt %, and lower than the 0.75 wt %.

These findings imply that higher QYs are achieved not only by optimizing the IET but also by increasing the population and radiative decay of the ^5^D_0_ manifold. Hence, to strategize luminescent films with enhanced performance, one should move beyond IET alone by also considering influences such as the structural properties of the complex embedded in polymers. This effect may indirectly influence population dynamics. For example, the simulations evinced a more compact arrangement of PVDF molecules around the complex, which induced a higher triplet state energy for the [Eu­(dbm)_4_]^−^/PVDF system compared to [Eu­(dbm)_4_]^−^/PMMA, thereby affecting the sensitization mechanism. Hence, manipulating the organic groups in the polymer surrounding Eu^III^ is crucial for the design of systems. Although the modeling protocol for the PVDF film targets the amorphous fraction, the sizable distortions needed to reproduce the experimental values unequivocally underscore the influence of the semicrystalline domains – an essential factor for shedding light on the material luminescence dynamics. Such occurrences are an illustrative example of how the proposed protocol can lead the way to understanding and predicting the capabilities of luminescent films. The notable alignment between the experimental and theoretical quantum yield values while maintaining a low computational cost endorses the suitability of the proposed protocol. In this sense, integrating QM/QM calculations with the modeling of Ln^III^ photophysical characteristics proves to be a valuable strategy for designing films with enhanced luminescence.

## Conclusions

In this study, an in-depth investigation of [Eu­(dbm)_4_]^−^ dispersed in PMMA and PVDF matrices was conducted using multilevel quantum mechanical methods to model the photophysical properties of Ln^III^-based compounds. An interesting outcome of the simulation was that the tighter packing of PVDF around the complex resulted in pronounced distortion of the ligand scaffold and Eu^III^ microssymmetry. These angular changes were the primary cause of the elevated Ω_2_ values, whereas substantial alterations in polarizability led to a 6-fold increase in Ω_4_. This increase can be attributed to the modifications of the coordination polyhedron of Eu^III^, which reduced Eu–O bond lengths. Considering these distortions in terms of spherical coordinates allowed us to estimate the energy transfer and back-energy transfer rates. A higher concentration of the complex in the composition boosted the ET and BET rates by an uneven factor. Intriguingly, the [Eu­(dbm)_4_]^−^/PVDF film displayed lower ET rates by an order of magnitude compared to [Eu­(dbm)_4_]^−^/PMMA, which reflected in the lower population of the ^5^D_0_ level for PVDF and consequently, reduced QY. This outcome underscores the predictive capability of our proposed methodology while shedding light on the mechanisms controlling the photophysical attributes of complex/polymer systems. These findings pave the way for materials development by strategically selecting systems where distortion occurs. Further studies aimed at detailing complex-polymer interactions via the composition of normal modes of these complexes are expected to offer deeper insights into the influence of the polymer on the photophysical attributes of these composites.

## Supplementary Material



## Notes

Further details of the theoretical methodology employed in this study, including the models and equations. The supporting materials also include experimental SEM and EDS analysis of the film. The outcomes harnessed by the system of ordinary differential equations to harvest the population of the emissive state are also shown (PDF)

## Abbreviations

QY: quantum yield
ET: energy transfer
BET: back-energy transfer
DFT: density functional theory
QM: quantum mechanics
SQM: semiempirical quantum mechanics
SP-SQM: self-parametrized semiempirical quantum mechanics
PMMA: poly­(methyl) methacrylate
PVDF: polyvinylidene fluoride
dbm: 1,3-diphenyl-1,3-propanedione

## References

[1] Gálico D. A., Kitos A. A., Ovens J. S., Sigoli F. A., Murugesu M. (2021). Lanthanide-Based
Molecular Cluster-Aggregates: Optical Barcoding and White-Light Emission
with Nanosized {Ln_20_} Compounds. Angew. Chem., Int. Ed..

[2] Gálico D. A., Murugesu M. (2021). Inside-Out/Outside-In Tunability in Nanosized Lanthanide-Based
Molecular Cluster-Aggregates: Modulating the Luminescence Thermometry
Performance via Composition Control. ACS Appl.
Mater. Interfaces.

[3] Calado C. M. S., Gálico D. A., Murugesu M. (2023). Composition Control
in Molecular Cluster-Aggregates: A Toolbox for Optical Output Tunability *via* Energy Transfer Pathways. ACS
Appl. Mater. Interfaces.

[4] Carneiro
Neto A. N., Teotonio E. E. S., de Sá G. F., Brito H. F., Legendziewicz J., Carlos L. D., Felinto M. C. F. C., Gawryszewska P., Moura R. T., Longo R. L., Faustino W. M., Malta O. L. (2019). Chapter 310-Modeling intramolecular
energy transfer in lanthanide chelates. A critical review and recent
advances. Handb. Phys. Chem. Rare Earths.

[5] Ferreira
da Rosa P. P., Kitagawa Y., Shoji S., Oyama H., Imaeda K., Nakayama N., Fushimi K., Uekusa H., Ueno K., Goto H., Hasegawa Y. (2022). Preparation of photonic
molecular trains via soft-crystal polymerization of lanthanide complexes. Nat. Commun..

[6] Thor W., Wu Y., Wang L., Zhang Y., Tanner P. A., Wong K.-L. (2021). Charging
and ultralong phosphorescence of lanthanide facilitated organic complex. Nat. Commun..

[7] Dalal A., Nehra K., Hooda A., Singh D., Kumar P., Kumar S., Malik R. S., Rathi B. (2023). Luminous lanthanide
diketonates: Review on synthesis and optoelectronic characterizations. Inorg. Chim. Acta.

[8] Sato S., Wada M. (1970). Relations between Intramolecular
Energy Transfer Efficiencies and
Triplet State Energies in Rare Earth β-diketone Chelates. Bull. Chem. Soc. Jpn..

[9] Costa I. F., Blois L., Paolini T. B., Assunção I. P., Teotonio E. E. S., Felinto M. C. F. C., Moura-Jr R. T., Longo R. L., Fastino W. M., Dias L. C., Malta O. L., Carneiro
Neto A. N., Brito H. F. (2024). Luminescence Properties of lanthanide
tetrakis complexes as molecular light emitters. Coord. Chem. Rev..

[10] Binnemans K. (2005). Chapter 225
- Rare-earth beta-diketonates. Handb. Phys.
Chem. Rare Earths.

[11] Pham Y. H., Trush V. A., Carneiro Neto A. N., Korabik M., Sokolnick J., Weselski M., Malta O. L., Amirkhanov V. M., Gawryszewska P. (2020). Lanthanide complexes with *N*-phosphorylated
carboxamide as UV converters with excellent emission quantum yield
and single-ion magnet behavior. J. Mater. Chem.
C.

[12] de
Freitas B. D., Onish B. S. D., Caixeta F. J., Bortoletto-Santos R., Garcia F. D. R., Massadeq Y., Ribeiro S. J. L. (2023). Green host urethanesil
based on castor oil doped with Eu^3+^ complex. Opt. Mater..

[13] Leite
Silva C. M. B., Bispo-Jr A. G., Lima S. A. M., Pires A. M. (2019). Eu^3+^ complex/polymer films for light-emitting diode applications. Opt. Mater..

[14] Beltrame A. C. F., Bispo-Jr A. G., Canisares F. S. M., Fernandes R. V., Laureto E., Lima S. A. M., Pires A. M. (2023). PMMA or
PVDF films
blended with β-diketonate tetrakis Eu^III^ or Tb^III^ complexes used as downshifting coatings of near-UV LEDs. Soft Matter.

[15] Ilmi R., Zhang D., Tensi L., Al-Sharji H., Al Rasbi N. K., Macchioni A., Zhou L., Wong W. Y., Raithby P. R., Khan M. S. (2022). Salts of Lanthanide­(III) Hexafluoroacetylacetonates
[Ln = Sm­(III), Eu­(III) and Tb­(III)] with Dipyridylammonium cations:
Synthesis, characterization, photophysical properties and OLED fabritcation. Dyes Pigm..

[16] Biju S., Freire R. O., Eom Y. K., Scopelliti R., Bünzli J. C. G., Kim H. K. (2014). A Eu^III^ Tetrakis­(β-diketonate)
Dimeric Complex: Photophysical Properties, Structural Elucidation
by Sparkle/AM1 Calculations, and Doping into PMMA Films and Nanowires. Inorg. Chem..

[17] Alam N., Mondal S., Hossain S. K. S., Sahoo S., Sarma D. (2023). Lanthanide-Directed
Luminescent “Soft” Coordination Polymer Gels: White
Light Emission, Anticounterfeiting, and Thin-Film-Based Sensing. ACS Appl. Eng. Mater..

[18] Dias L. M. S., Fu L., Pereira R. F. P., Carneiro Neto A. N., De Zea Bermudez V., André P. S., Ferreira R. A. S. (2024). Envolving photonic
authentication with sustainable luminescent smart e-tags. FlexMat.

[19] Gil-Kowalczyk M., Łyszczek R., Jusza A., Piramidowicz R. (2021). Thermal, Spectroscopy
and Luminescent Characterization of Hybrid PMMA/Lanthanide Complex
Materials. Materials.

[20] Francisco L. H. C., Felinto M. C. F. C., Brito H. F., Teotonio E. E. S., Malta O. L. (2019). Development of highly
luminescent PMMA films doped
with Eu^3+^ β-diketonate coordinated on ancillary ligand. J. Mater. Sci.: Mater. Electron..

[21] Assunção I. P., Blois L., Cauli F. P., Felinto M. C. F. C., Malta O. L., Brito H. F. (2024). Luminescence
under UV­(A, B and C)
and sunlight exposure of tetrakis Tb^3+^ carboxylate complexes
doped in different polymers. J. Alloys Compd..

[22] Caixeta F. J., Saraiva L. F., Freitas B. D., Onishi B. S., Santagneli S. H., Bortoletto-Santos R., Pires A. M., Ribeiro S. J. L. (2025). Spectroscopic
and theoretical tools unravel the thermally-stabilized behavior of
Eu^3+^-Based complex incorporated in sustainable urethanesil
film. Chem. - Asian J..

[23] Saraiva L. F., Bispo-Jr A. G., Lima S. A. M., Pires A. M. (2023). Unrevealing
the
opto-structural features of luminescente polymeric films containing
Eu^III^-doped phosphors through spectroscopic and theoretical
perspectives. J. Mater. Chem. C.

[24] McCarver G. A., Hinde R. J., Vogiatzis K. D. (2020). Selecting
Quantum-Chemical Methods
for Lanthanide-Containing Molecules: A Balance between Accuracy and
Efficiency. Inorg. Chem..

[25] Diogenis I. M. S., Bispo A. G., Pirovani R. V., Saraiva L. F., Gozzo F. C., Correia C. R. D., Mazali I. O., Nome R. A., Sigoli F. A. (2024). Towards opto-structural
parameters to enhance the circularly
polarized luminescence brightness of Eu^III^ β-diketone
complexes with chiral auxiliary ligands. J.
Mater. Chem. C.

[26] Saraiva L. F., Bispo A. G., Lima S. A. M., Pires A. M. (2023). Eu^3+^-activated SrY_2_O_4_:Ce^4+/3+^ red-phosphor for WLEDs: Structural
and luminescence insights from
experimental and theoretical perspectives. J.
Alloys Compd..

[27] Saraiva L. F., Carneiro Neto A. N., Bispo-Jr A. G., Quintano M. M., Kraka E., Carlos L. D., Lima S. A. M., Pires A. M., Moura R. T. (2025). Role of Vibronic Coupling for the Dynamics of Intersystem
Crossing in Eu^3+^ Complexes: An avenue for Brighter Compounds. J. Chem. Theory Comput..

[28] Plett C., Grimme S. (2023). Automated and Efficient Generation
of General Molecular
Aggregate Structures. Angew. Chem., Int. Ed..

[29] Momany F. A., Rone R. (1992). Validation of the general
purpose QUANTA R3.2/CHARMm force field. J. Comput.
Chem..

[30] MacKerell A. D., Banavali N., Foloppe N. (2000). Development and current
status of the CHARMM force field for nucleic acids. Biopolymers.

[31] Salomon-Ferrer R., Case D. A., Walker R. C. (2013). An overview
of the Amber biomolecular
simulation package. WIREs Comput. Mol. Sci..

[32] Spicher S., Grimme S. (2020). Robust Atomistic Modeling
of Materials, Organometallic,
and Biochemical Systems. Angew. Chem., Int Ed..

[33] Bannwarth C., Ehlert S., Grimme S. (2019). GFN2-xTB–An Accurate and Broadly
Parametrized Self-Consistent Tight-Binding Quantum Chemical Method
with Multipole Electrostatic and Density-Dependent Dispersion Contributions. J. Chem. Theory Comput..

[34] Christensen A. S., Kubar T., Cui Q., Elstner M. (2016). Semiempirical Quantum
Mechanical Methods for Noncovalent Interactions for Chemical and Biochemical
Applications. Chem. Rev..

[35] Plett C., Katbashev A., Ehlert S., Grimme S., Bursch M. (2023). ONIOM meets
xtb: efficient, accurate, and robust multi-layer simulations across
the periodic table. Phys. Chem. Chem. Phys..

[36] Judd B. R. (1962). Optical
Absorption Intensities of Rare-Earth Ions. Phys.
Rev..

[37] Ofelt G. S. (1962). Intensities
of Crystal Spectra of Rare-Earth Ions. J. Chem.
Phys..

[38] Malta O. L. (2008). Mechanism
of non-radiative energy transfer involving lanthanides ions revisited. J. Non-Cryst. Solids.

[39] Malta O. L. (1997). Ligand-rare
Earth ion energy transfer in coordination compounds: A theoretical
approach. J. Lumin..

[40] e
Silva F. R. G., Malta O. L. (1997). Calculation of the ligand-lanthanide
ion energy transfer rate in coordination compounds: contribution exchange
interactions. J. Alloys Compd..

[41] Shyichuk A., Moura R. T., Carneiro Neto A. N., Runowski M., Zarad M. S., Szczeszak A., Lis S., Malta O. L. (2016). Effects of Dopant Addition on Lattice and Luminescence
Intensity Parameters of Eu­(III)-Doped Lanthanum Orthovanadate. J. Phys. Chem. C.

[42] El-Hady M. M., Morshidy H. Y., Hassan M. A. (2023). Judd-Ofelt analysis,
optical and
structural features of borate glass doped with erbium oxide. J. Lumin..

[43] Bispo-Jr A. G., Saraiva L. F., Lima S. A. M., Pires A. M., Mazali I. O., Sigoli F. A. (2025). Lanthanide coordination
polymers
as luminescent thermometers: integrating theoretical modeling with
experimental analysis to tune the thermal response. J. Mat Chem. C.

[44] Leite
Silva C. M. B., Bispo-Jr A. G., Canisares F. S. M., Castilho S. A., Lima S. A. M., Pires A. M. (2019). Eu^3+^-tetrakis
β-diketonate complexes for solid-state lighting application. Luminescence.

[45] Zhou Y., Liu W., Tan B., Zhu C., Ni Y., Fang L., Lu C., Xu Z. (2021). Crystallinity and β Phase Fraction of PVDF in
Biaxiality Stretched PVDF/PMMA Films. Polymers.

[46] Neese F. (2022). Software update:
The ORCA program system – Version 5.0. Wires Comp. Mol. Sci..

[47] Grimme S., Hansen A., Ehlert S., Mewes J.-M. (2021). r^2^SCAN-3c:
A “Swiss army knife” composite electronic-structure
method. J. Chem. Phys..

[48] Müller M., Hansen A., Grimme S. (2023). ωB97X-3c: A composite range-separated
hybrid DFT method with a molecule-optimized polarized valence double-ζ
basis set. J. Chem. Phys..

[49] Weigend F., Ahlrichs R. (2005). Balanced basis sets of split valence, triple zeta valence
and quadruple zeta valence quality for H to Rn: Design and assessment
of accuracy. Phys. Chem. Chem. Phys..

[50] Dolg M., Stoll H., Savin A., Preuss H. (1989). Energy-adjusted pseudopotentials
for the rare earth elements. Theoret. Chim.
Acta.

[51] Dolg M., Stoll H., Preuss H. (1993). A combination
of quasirelativistic
pseudopotential and ligand field calculations for lanthanoid compounds. Theoret. Chim. Acta.

[52] Chai J.-D., Head-Gordon M. (2008). Long-range corrected hybrid density functionals with
damped atom-atom dispersion corrections. Phys.
Chem. Chem. Phys..

[53] Martínez L., Andrade R., Birgin E. G., Martínez J. M. (2009). PACKMOL:
A package for Building initial configurations for molecular dynamics
simulations. J. Comput. Chem..

[54] Grimme S. (2019). Exploration
of Chemical Compound, Conformer, and Reaction Space with Meta-Dynamics
Simulations Based on Tight-Binding Quantum Chemical Calculations. J. Chem. Theory Comput..

[55] Moura R. T., Carneiro Neto A. N., Longo R. L., Malta O. L. (2016). On the calculation
and interpretation of covalency in the intensity parameters of 4f-4f
transitions in Eu^3+^ complexes based on the chemical bond
overlap polarizability. J. Lumin..

[56] Moura R. T., Quintano M., Santos C. V., Albuquerque V. A. C. A., Aguiar E. C., Kraka E., Carneiro Neto A. N. (2022). Featuring
a new computational protocol for the estimation
of intensity and overall quantum yield in lanthanide chelates with
applications to Eu­(III) mercapto-triazole Schiff base ligands. Opt. Mater.:X.

[57] Kraka E., Quintano M., La Force H. W., Antonio J. J., Freindorf M. (2022). The Local
VIbrational Mode Theory and Its Place in the Vibrational Spectroscopy
Arena. J. Phys. Chem. A.

[58] Santos-Jr C. V., Aguiar E. C., Carneiro
Neto A. N., Moura R. T. (2023). Adaptive guided
stochastic optimzation: A novel approach for fitting
the theoretical intensity parameters for lanthanide compounds. Opt. Mater.:X.

[59] Moura R. T., Carneiro
Neto A. N., Aguiar E. C., Santos-Jr C. V., de Lima E. M., Faustino W. M., Teotonio E. E. S., Brito H. F., Felinto M. C. F. C., Ferreira R. A. S., Carlos L. D., Longo R. L., Malta O. L. (2021). JOYSPectra: A web platform for luminescence of lanthanides. Opt. Mater.:X.

[60] Malta O. L., Gonçalves e Silva F. R. (1998). A theoretical
approach to intramolecular
energy transfer and emission quantum yields in coordination compounds
of rare earth ions. Spectrochim. Acta, Part
A.

[61] Carneiro
Neto A. N., Mamontova E., Botas A. M. P., Brites C. D. S., Ferreira R. A. S., Rouquette J., Guari Y., Larionova J., Long J., Carlos L. D. (2022). Rationalizing
the Thermal Response of Dual-Center Molecular Thermometers: The Example
of na Eu/Tb Coordination Complexes. Adv. Opt.
Mater..

[62] Bünzli J.-C. (2015). On the
design of highly luminescent lanthanide complexes. Coord. Chem. Rev..

[63] Fang M., Carneiro Neto A. N., Fu L., Ferreira R. A. S., deZeaBermudez V., Carlos L. D. (2022). A Hybrid Materials Approach for Fabricating Efficient
WLEDs Based on Di-Ureasils Doped with Carbon Dots and a Europium Complex. Adv. Mater. Technol..

[64] Kaappa S., Malola S., Häkkinen H. (2018). Point Group
Analysis of the Electronic
Structure of Bare and Protected Metal Nanocrystals. J. Phys. Chem. A.

[65] Srikanth A., Abrams C. F. (2019). Effect of molecular
packing and hydrogen bonding on
the properties of epoxy-amido amine systems. Comput. Mater. Sci..

[66] Wang S., Yuan K., Wang K., Chen W., Yamada K., Barkley D., Koga T., Hong Y.-L., Miyoshi T. (2019). Intramolecular
and intermolecular packing in polymer crystallization. Macromolecules.

[67] de
Souza J. M., Alves S., Sá G. F., Azevedo W. M. (2002). Doped polymers with Ln­(III) complexes: simulation and
control of light colors. J. Alloys Compd..

[68] Mahmood Z., Aleem A. R., Sial Q. A., Wang G., Usman M., Khokhar W. A., Wei C., Liu X., Ding W. (2024). Eu^3+^ embedded hybrid fluorescent membrane
for Ultrasensitive and efficient
sensing of Cu^2+^ in aqueous media. Dyes Pig..

[69] Hayashi J., Shoji S., Kitagawa Y., Hasegawa Y. (2022). Amorphous lanthanide
complex for organic luminescent materials. Coord.
Chem. Rev..

[70] Bispo-Jr A. G., Oliveira N. A., Cardoso C. X., Lima S. A. M., Job A. E., Osorio-Román I. O., Danna C. S., Pires A. M. (2018). Red-light
emitting polymer composite based on PVDF membranes and Europium phosphor
using Buriti Oil as plasticizer. Mater. Chem.
Phys..

[71] Ćirić A., Marciniak L., Dramicanin M. D. (2022). Self-referenced method for the Judd-Ofelt
parametrisation of the Eu^3+^ excitation spectrum. Sci. Rep..

[72] Blois L., Carneiro Neto A. N., Longo R. L., Costa I. F., Paolini T. B., Brito H. F., Malta O. L. (2022). On the experimental Determination
of 4*f*-4*f* Intensity Parameters from
the Emission Spectra of Europium­(III) Compounds. Opt. Spectrosc..

[73] Hehlen M. P., Brik M. G., Krämer K. W. (2013). 50th anniversary
of the Judd-Ofelt
theory: An experimentalist’s view of the formalism and its
application. J. Lumin..

[74] Binnemans K. (2015). Interpretation
of europium­(III) spectra. Coord. Chem. Rev..

[75] Thor W., Carneiro Neto A. N., Moura R. T., Wong K.-L., Tanner P. A. (2024). Europium­(III)
coordination chemistry: structure, spectra
and hypersensitivity. Coord. Chem. Rev..

[76] Malta O. L., Carlos L. D. (2003). Intensities of 4f-4f
transitions in glass materials. Quim. Nova.

[77] Malta O. L., Batista H. J., Carlos L. D. (2002). Overlap polarizability of a Chemical
bond: a scale of covalency and application to lanthanide compounds. Chem. Phys..

[78] Suta M., Cimpoesu F., Urland W. (2021). The angular overlap
model of ligand
field theory for f elements: An intuitive approach building bridges
between theory and experiment. Coord. Chem.
Rev..

[79] Wybourne B. G., Meggers W. F. (1965). Spectroscopic Properties of Rare Earths. Phys. Today.

[80] Hasegawa Y., Kitagawa Y. (2022). Luminescent lanthanides coordination polymers with
transformative energy transfer processes for physical and chemical
sensing applications. J. Photochem. Photobiol.,
C.

[81] Tanner P. A., Zhou L., Duan C., Wong K.-L. (2018). Misconceptions in
electronic energy transfer: bridging the gap between chemistry and
physics. Chem. Soc. Rev..

[82] Kasprzycka E., Carneiro Neto A. N., Trush V. A., Malta O. L., Jerzykiewicz L., Amirkhanov V. M., Legendziewicz J., Gawryszewska P. (2022). Spectroscopic
aspects for the Yb^3+^ coordination compound with a large
energy gap between the ligand Yb^3+^ excited states. Spectrochim. Acta, Part A.

[83] Saraiva L. F., Bispo A. G., Costa A. L., Sigoli F. A., Lima S. A. M., Pires A. M. (2025). Dimensionality reduction
expands
the frontiers of lanthanide luminescence thermometry beyond single-parametric
thermal sensing. Chem. Mater..

[84] Blois L., Carneiro Neto A. N., Malta O. L., Brito H. F. (2022). The role of the
Eu^3+7^F_1_ level in the direct sensitization of
the ^5^D_0_ emitting level through intramolecular
energy transfer. J. Lumin..

[85] Ramalho J. F. C. B., Carneiro Neto A. N., Carlos L. D., André P. S., Ferreira R. A. S. (2022). Lanthanides for the new Generation of optical sensing
and internet of things. Handb. Phys. Chem. Rare
Earths.

[86] Tanner P. A., Thor W., Zhang Y., Wong K.-L. (2022). Energy Transfer
Mechanism and Quantitative Modeling of Rate from an Antenna to a Lanthanide
ion. J. Phys. Chem. A.

