# Supplementary material for: Unfolding the Photophysical Behavior of Luminescent Polymeric Films Containing β‑Diketonate Tetrakis EuIII Complexes via Multilayer Quantum Mechanics
Source: ACS Omega. 2025 Jul 12;10(28):30563–75. doi: 10.1021/acsomega.5c02434 (PMC12290699; doi:10.1021/acsomega.5c02434)
Supplement: Supplementary file 1 [file ao5c02434_si_001.pdf]

Supplementary Information

Unfolding the Photophysical Behavior of Luminescent Polymeric Films Containing β-Diketonate Tetrakis Eu^{III} Complexes via Multilayer Quantum Mechanics

Leonardo F. Saraiva[O †,‡,§], Ariane C. F. Beltrame[O †, ‡,ǁ], Airton G. Bispo-Jr[⊥]*, Felipe S. M. Canisares[⊥], Albano N. Carneiro Neto[§], Renaldo T. Moura Jr. [#]*, E. Kraka[∇], Sergio A. M. Lima[†,‡], and Ana M. Pires[†,‡]*

[O] These authors contributed equally to this study.

[†] Department of Chemistry and Biochemistry, School of Science and Technology, São Paulo State University (UNESP), 19060-900 Presidente Prudente, Brazil.

[‡] Institute of Biosciences, Humanities and Exact Sciences, Department of Chemistry and Environmental Sciences, São Paulo State University (UNESP), 15054-000 São José do Rio Preto, Brazil.

State University (UNESP), 15054-000 São José do Rio Preto, Brazil.

[§] Department of Physics, University of Aveiro, 3810-193 Aveiro, Portugal.

[ǁ] Department of Sustainable Development and Ecological Transition (DISSTE), University of Eastern Piedmont "A. Avogadro", 13100 Vercelli, Italy.

[⊥] Institute of Chemistry, University of São Paulo (USP), 05508-900 São Paulo, Brazil.

[#] Academic Unit of Cabo de Santo Agostinho, Federal Rural University of Pernambuco (UFRPE), 54518-430 Cabo de Santo Agostinho, Brazil.

[∇] Department of Chemistry (Computational and Theoretical Chemistry Group), Southern Methodist University (SMU), 75725 Dallas, Texas, United States.

Table of contents

Supplementary note S1 – Microscopy analysis 2

Supplementary note S2 – Theoretical details 2

Theoretical intensity parameters 2

Intramolecular energy transfer 3

Matrix element calculation 5

Supplementary note S3 – Structural properties 6

Geometry optimization 6

Scanning electron microscopy and chemical mapping 9

Supplementary Note S4 – Judd-Ofelt intensity parameters 10

Supplementary note S5 – Electronic structure 19

Supplementary note S6 – Energy transfer and populations 21

References 27

Supplementary note S1 – Microscopy analysis

The scanning electron microscopy (SEM) analysis was conducted on a Hitachi model SU3800 scanning-electron microscope with a detector of secondary and backscattering electrons in a high vacuum and at a constant temperature. The films were previously coated with a thin gold layer by using a sputtering QUORUM, Q 150R ES model. Given the resolution of the equipment (0.5 at.%), only 1 wt.% films were measured.

Supplementary note S2 – Theoretical details

Theoretical intensity parameters

To calculate the theoretical intensity parameters, we used the Eq. S1 – S5. Within these equations, the forced electric dipole (FED) is derived from the original Judd-Ofelt theory[1,2] in the framework of the simple overlap model (SOM)[3] and bond overlap model (BOM)[4].

$$\Omega_\lambda^{theo} = (2\lambda+1)\sum_{t,p}\frac{\left|B_{\lambda tp}\right|^2}{2t+1}, B_{\lambda tp} \;=\; B_{\lambda tp}^{FED} + B_{\lambda tp}^{DC} \qquad (S1)$$

$$B_{\lambda tp}^{FED} = \frac{2}{\Delta E}\langle r^{t+1}\rangle \Theta(t,\lambda)\gamma_p^t \qquad (S2)$$

$$B_{\lambda tp}^{DC} = \left[-\frac{(\lambda+1)(2\lambda+3)}{(2\lambda+1)}\right]^{1/2}\langle r^\lambda\rangle\langle f\|C^{(\lambda)}\|f\rangle\Gamma_p^t\delta_{t,\lambda+1} \quad (S3)$$

$$\gamma_p^t = \left(\frac{4\pi}{2t+1}\right)^{1/2}\left(\sum_j e^2\rho_j g_j\left(2\beta_j\right)^{t+1}\frac{Y_{p,j}^{t*}}{R_j^{t+1}}\right) \qquad (S4)$$

$$\Gamma_p^t = \left(\frac{4\pi}{2t+1}\right)^{1/2}\left(\sum_j\left[\left(2\beta_j\right)^{t+1}\alpha_{OP,j} + \alpha_j'\right]\frac{Y_{p,j}^{t*}}{R_j^{t+1}}\right) \qquad (S5)$$

In these equations, the numerical factors ($\Theta(t,\lambda)$), establish the relationship between the *f-g* and *f-d* interconfigurational transitions and 4*f* radial integrals[5]. The specific values are: $\Theta(1,2) = -0.17$, $\Theta(3,2) = 0.34$, $\Theta(3,4) = 0.18$, $\Theta(5,4) = -0.24$, $\Theta(5,6) = -0.24$, and $\Theta(7,6) = 0.24$[5]. Additionally, ΔE represents the average energy denominator method[6]. The overlap charge associated with the coordinating atom and the Ln^{III} is described by the charge factor (g_j) and overlap integral (ρ_j), while $\beta_j =$

$\left(1 \pm \rho_j\right)^{-1}$[4]. The site environment symmetry is treated by the sum over j with conjugated spherical harmonics ($Y_{p,j}^{t*}$). As the spherical harmonics depend on the coordinates of the atoms, inducing structural changes alters these harmonics providing deviations in the overall values. Alternatively, Eq. (S3) expresses the contribution of dynamic coupling (DC) within the framework of the bond overlap model (BOM), which characterizes the polarizability of the ligand through $\alpha_{OP,j}$, and α_j'. The former represents the bond overlap polarizability, while the latter denotes the effective polarizability og the ligand[4]. These quantities were obtained from the *JoySpectra* platform[7] using the charge factors (g_j), bond overlap polarizability ($\alpha_{OP,j}$) and effective polarizability (α'), represented by Eq. S6 – S7, while the α' was determined from the fitting procedure[8].

$$g_j = R_j \sqrt{\frac{k_j}{2\Delta\varepsilon_j}} \qquad (S6)$$

$$\alpha_{OP,j} = \frac{e^2 {\rho_j}^2 R_j^2}{2\Delta\varepsilon_j} \qquad (S7)$$

In these equations, R_j is the bond length, $\Delta\varepsilon_j$ is the first excitation energy associated with Ln–L ligating atom species, and k_j is the force constant of the Ln – L bond, which can be determined from the local vibrational modes[9]. The electron charge is represented by e while ρ_j is the overlap integral. The values of $\Delta\varepsilon_j$ and ρ_j were extracted from reference [10] using an exponential fit.

Intramolecular energy transfer

The rates of intramolecular energy transfer (IET) from the excited states S_1 and T_1 of the antenna ligand to the Eu^{III} ion can be determined considering the dipole-dipole (W_{d-d}), dipole-multipole (W_{d-m}), and exchange mechanism (W_{ex}), as written in equations S8 – S10, where S_d^{Ln}, S_λ^{Ln}, S_{ex}^{L}, and S_{ex}^{Ln} are hidden in equations S11 – S14 for clarity[11,12,13,14].

$$W_{d-d} = \frac{2\pi}{\hbar}\left(\frac{S_d^L}{G}\frac{S_d^{Ln}}{R_L^6}\right)F \qquad (S8)$$

$$W_{d-m} = \frac{2\pi}{\hbar}\left[\frac{S_d^L}{G}\left(\sum_\lambda \frac{S_\lambda^{Ln}}{\left(R_L^{\lambda+2}\right)^2}\right)\right]F \qquad (S9)$$

$$W_{ex} = \frac{2\pi}{\hbar}\left(\frac{S_{ex}^{L}}{G}\frac{S_{ex}^{Ln}}{R_L^4}\right)F \qquad (S10)$$

$$S_d^{Ln} = \frac{2e^2(1-\sigma_1)^2}{(2J+1)}\sum_{\lambda} \Omega_{\lambda}^{FED}\left\langle \psi'J'\middle|\middle|U^{(\lambda)}\middle|\middle|\psi J\right\rangle^2 \qquad (S11)$$

$$S_{\lambda}^{Ln} = \frac{e^2(1-\sigma_{\lambda})^2(\lambda+1)}{(2J+1)}\langle r^{\lambda}\rangle^2\left\langle f\middle|\middle|C^{(\lambda)}\middle|\middle|f\right\rangle^2\left\langle \psi'J'\middle|\middle|U^{(\lambda)}\middle|\middle|\psi J\right\rangle^2 \qquad (S12)$$

$$S_{ex}^{L} = \frac{4}{3}\frac{(1-\sigma_0)^2}{(2J+1)}e^2\langle \psi'J'||S||\psi J\rangle^2 \qquad (S13)$$

$$S_{ex}^{Ln} = e^2\sum_{m}\left|\langle\varphi|\textstyle\sum_j \mu_z(j)s_m(j)\,|\varphi *\rangle\right|^2 \qquad (S14)$$

In all equations, R_L represents the distance between the donor and acceptor states, which is obtained from the excited state calculation. The quantity Ω_{λ}^{FED} denotes the intensity parameters regarding only the forced electric–dipole contribution. The terms $\left\langle \psi'J'\middle|\middle|U^{(\lambda)}\middle|\middle|\psi J\right\rangle^2$ were obtained from reference [15], while the squared spin-operator matrix elements, $\langle \psi'J'||S||\psi J\rangle^2$ were calculated using free-ion wave functions within the intermediate coupling scheme[16,17] (**Table S1**). S_L corresponds to the dipole strength of the ligand transition involved in the IET process, with values of 10^{-36} and 10^{-40} esu^2 cm^2 for S_1 and T_1, respectively[11]. Factor *G* represents the state degeneracy (equal 1 for S_1 and 3 for T_1), while the shielding factors $(1-\sigma_k)$ are also considered[18].

For lanthanide-based systems, the IET process is assumed to be non-resonant[12]. Consequently, all equations require an energy mismatch factor (F), represented in equation S15. Here, we consider that the bandwidth of the ligands ($\gamma_L \approx 3000\ cm^{-1}$) is much larger than that of the lanthanides ($\gamma_{Ln} \approx 300\ cm^{-1}$), $\gamma_L \gg \gamma_{Ln}$[12]. The parameter δ represents the maximum energy difference between the donor state (D) and the acceptor state of the lanthanide ion, $\delta = E_D - E_{Ln}$. Forward energy transfer (W), therefore, can be obtained by summing all mechanisms (Equation S16)[14].

$$F = \frac{G(\delta,T)}{\hbar\gamma_L}\sqrt{\frac{\ln(2)}{\pi}}\,e^{-\left(\frac{\delta}{\hbar\gamma_L}\right)^2\ln(2)}, \qquad with\ G(\delta,T) = \begin{cases} 1\ if\ \delta \geq 0 \\ e^{\left(\frac{\delta}{k_B T}\right)}\ if\ \delta < 0 \end{cases} \qquad (S15)$$

$$W = W_{d-d} + W_{d-m} + W_{ex} \qquad (S16)$$

The backward energy transfer rates (W^b), that is, the energy returned from the acceptor to the donor state, can be obtained using the same equations, where k_B is the Boltzmann constant with a value of 1.3806×10^{-23} J K^{-1}, and T is the temperature.

Matrix element calculation

The matrix elements of the spin (S) operator between the states $\psi LSJ \longrightarrow \psi' L' S' J'$, which are relevant in the exchange mechanism of the intermolecular energy transfer (IET), can be computed within the framework of intermediate coupling scheme using the wave function for free ions (Eq. S17)[16,17].

$$\langle \psi LSJ || S || \psi' L' S' \rangle = \sum_i a_i \langle \psi LSJ || S || \psi' L' S' \rangle_i \quad (S17)$$

In our study, these matrix elements were expanded as a summation over the product of eigenfunctions $|\xi LSJ\rangle$ and $|\xi L' S' J'\rangle$, where the coefficients a_i are given by $\langle \xi LSJ | \xi L' S' J' \rangle$, as described in Eq. S18.

$$\langle \psi LSJ || S || \psi' L' S' \rangle = (-1)^{L+S+J'+1}[(2J+1)(2J'+1)S(S+1)(2S+1)]^{1/2} \begin{Bmatrix} S & J & L \\ J' & S & 1 \end{Bmatrix} \quad (S18)$$

Here, $\begin{Bmatrix} j_1 & j_2 & j_3 \\ j_4 & j_5 & j_6 \end{Bmatrix}$ represents the Wigner 6-j symbols, and their subsets (j_1, j_2, j_3), (j_1, j_5, j_6), (j_4, j_2, j_6), and (j_4, j_5, j_3) must satisfy the triangle rule in order to the 6-j symbols have non-zero values. The results of our calculation are presented in **Table S1**, which provides the values of the reduced matrix elements for Eu^{III} employed in this work.

Table S1. Values of squared reduced matrix elements for Eu^{III} ion. The values of $\langle \psi' J' || U^{(\lambda)} || \psi J \rangle^2$ were taken from ref. [15], while the $\langle \psi' J' || S || \psi J \rangle^2$ were calculated in this work.

Transition	$\langle \psi' J' \|\| U^{(2)} \|\| \psi J \rangle^2$	$\langle \psi' J' \|\| U^{(4)} \|\| \psi J \rangle^2$	$\langle \psi' J' \|\| U^{(6)} \|\| \psi J \rangle^2$	$\langle \psi' J' \|\| S \|\| \psi J \rangle^2$
**$^7F_0 \rightarrow {}^5D_0$**	8.0×10^{-6} *	0	0	0
**$^7F_0 \rightarrow {}^5D_1$**	0	0	0	2.73×10^{-2}
**$^7F_0 \rightarrow {}^5D_2$**	0.0008	0	0	0
**$^7F_0 \rightarrow {}^5G_6$**	0	0	0.0153	0
**$^7F_0 \rightarrow {}^5D_2$**	0	0	0.0037	0
**$^7F_0 \rightarrow {}^5D_4$**	0	0.0011	0	0
**$^7F_1 \rightarrow {}^5D_0$**	0	0	0	0.117
**$^7F_1 \rightarrow {}^5D_1$**	0.0025	0	0	2.81×10^{-5}
**$^7F_1 \rightarrow {}^5D_2$**	0	0	0	4.58×10^{-3}
**$^7F_1 \rightarrow {}^5D_3$**	0.0004	0.0012	0	0

$^7F_1 \rightarrow {}^5L_6$	0	0	0.0091	0
$^7F_1 \rightarrow {}^5L_7$	0	0	0.0181	0
$^7F_1 \rightarrow {}^5G_2$	0	0	0	1.28×10^{-2}
$^7F_1 \rightarrow {}^5G_3$	0.0002	0.0012	0	0
$^7F_1 \rightarrow {}^5G_6$	0	0	0.0049	0
$^7F_1 \rightarrow {}^5G_5$	0	0.0004	0.0097	0

* considering the J-mixing effect of 5%.

Supplementary note S3 – Structural properties

Geometry optimization

In the realm of the structure of the complex, the geometry optimization of the isolated complex, as well as the complex in the presence of polymer (found in Fig 1 of the main text), needs to be studied. In this sense, the degree of distortion can be qualitatively assessed by the root mean square deviation (RMSD) described in Eq. S19, which is calculated by neglecting translations between the center of mass of the molecule to reduce inaccuracies. In Eq. S19, v_i is the atomic coordinate vector related to the analyzed molecule taking Eu^{III} as reference, while w_i is the atomic coordinate vector related to the reference structure. Therefore, if one wants to analyze the distortion between PMMA and PVDF, one of them is taken as reference.

$$RMSD = \sqrt{\frac{1}{n}\sum_{i=1}^{n}\|v_i - w_i\|^2} = \sqrt{\frac{1}{n}\sum_{i=1}^{n}\left((v_{ix} - w_{ix})^2 + \left(v_{iy} - w_{iy}\right)^2 + (v_{iz} - w_{iz})^2\right)} \quad (S19)$$

Table S2. Mean values of bond length for the atoms coordinated to Eu^{III} in the three studied environments: $[Eu(dbm)_4]^-$, $[Eu(dbm)_4]^-$/PMMA and $[Eu(dbm)_4]^-$/PVDF. All values were obtained through geometry optimization. The ground-state conformer was used in this analysis.

$[Eu(dbm)_4]^{-S}$	Bond length / Å		
	**Isolated**	**PMMA**	**PVDF**
**Eu–O(1)**	2.530	2.531	2.469
**Eu–O(2)**	2.533	2.527	2.457
**Eu–O(3)**	2.529	2.515	2.441
**Eu–O(4)**	2.531	2.524	2.458
**Eu–O(5)**	2.529	2.521	2.462
**Eu–O(6)**	2.531	2.530	2.459
**Eu–O(7)**	2.529	2.534	2.458
**Eu–O(8)**	2.532	2.527	2.482

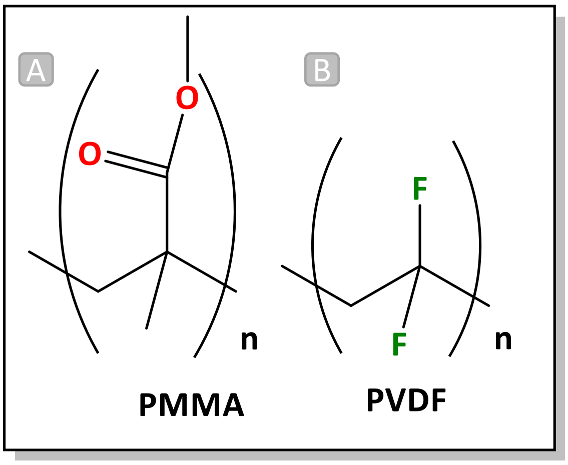

Fig S1. Structural fórmula of (a) PMMA and (b) PVDF polymers.

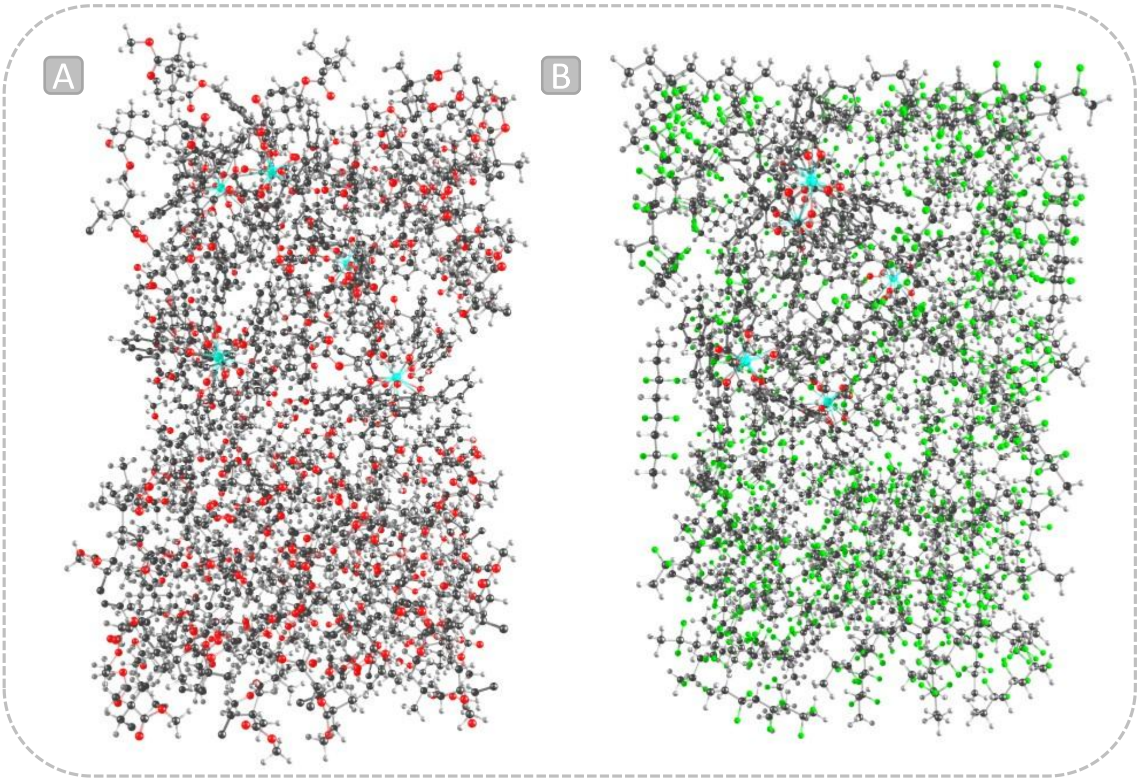

Fig S2. Simulated structures of the complex/polymer film by spawning 200 polymer motieties around the five units of $[Eu(dbm)_4]^-$ highlighting the packing. (a) $[Eu(dbm)_4]^-$/PMMA and (b) $[Eu(dbm)_4]^-$/PVDF. The color code is used as follows: cyan = europium, gray = carbon, green = fluorine, red = oxygen, and white = hydrogen.

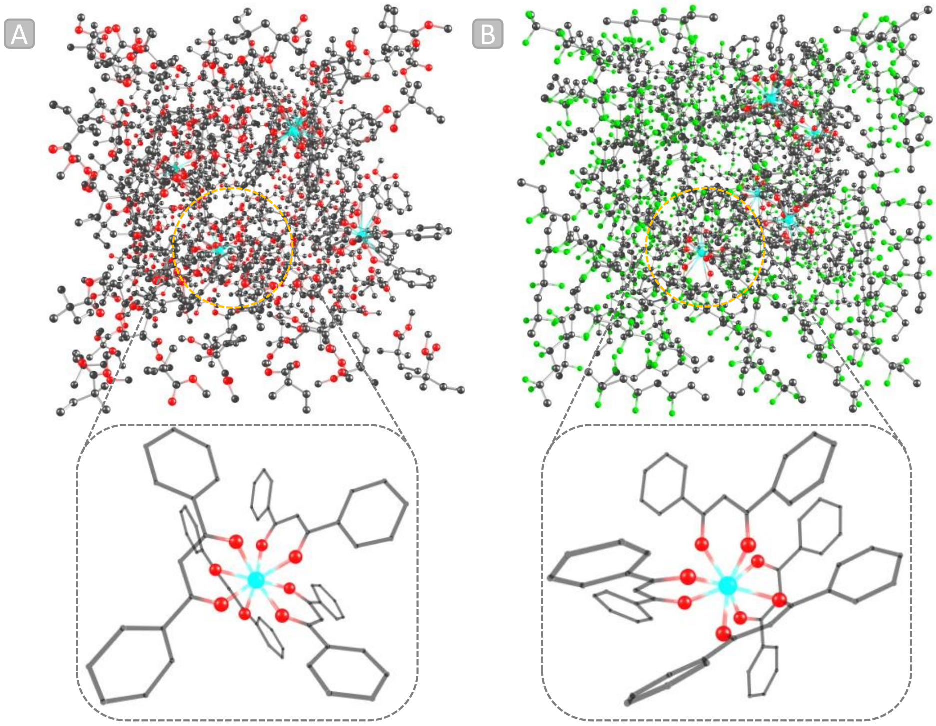

Fig S3. Ground-state QM/QM optimizations of $[Eu(dbm)_4]^-$ in (a) PMMA and (b) PVDF. The complex was treated at the high-level (DFT) and the polymer at the low-level (GFN2-xTB). The hydrogen atoms were hidden for clarity, and the color code is as follows: cyan = europium, gray = carbon, green = fluorine, and red = oxygen.

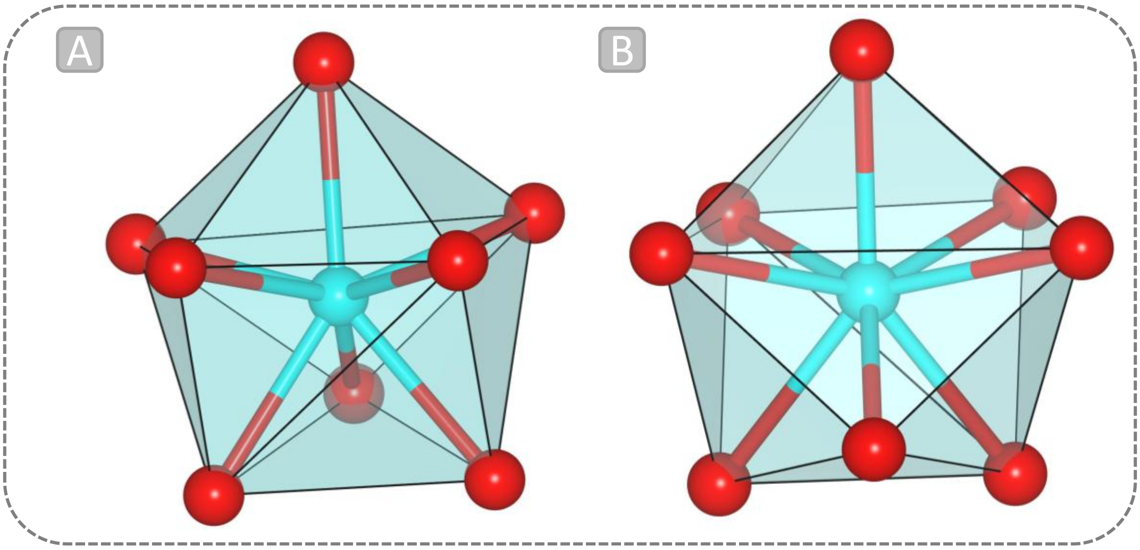

Fig S4. Coordination polyhedron of $[Eu(dbm)_4]^-$ in (a) PMMA and (b) PVDF.

Scanning electron microscopy and chemical mapping

The SEM images (**Fig S5**) reveal a uniform PMMA film (item a) punctuated by sparsely distributed surface particles. These inhomogeneities most plausibly originate from imperfections introduced during solvent evaporation or from the phase-segregation of trace residual complexes – a phenomenon previously reported by Beltrame et al[19]. In sharp contrast, the PVDF film (**Fig S5b**) displays the hallmark spherulitic agglomerates of PVDF membranes. These spherulites interconnected by narrow neck-like bridges, reflect the crystallization-driven lamellar impingement that typifies this polymer solidification behavior[20].

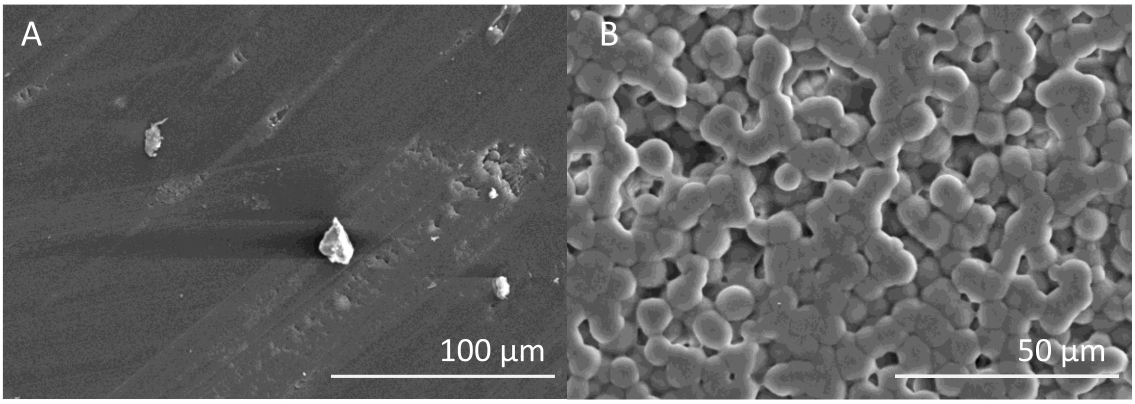

Fig S5. Scanning electron microscopy images of 1 wt.% of Eu-complex in (A) PMMA and (b) PVDF films. The films were prepared with the same procedure documented in reference [19].

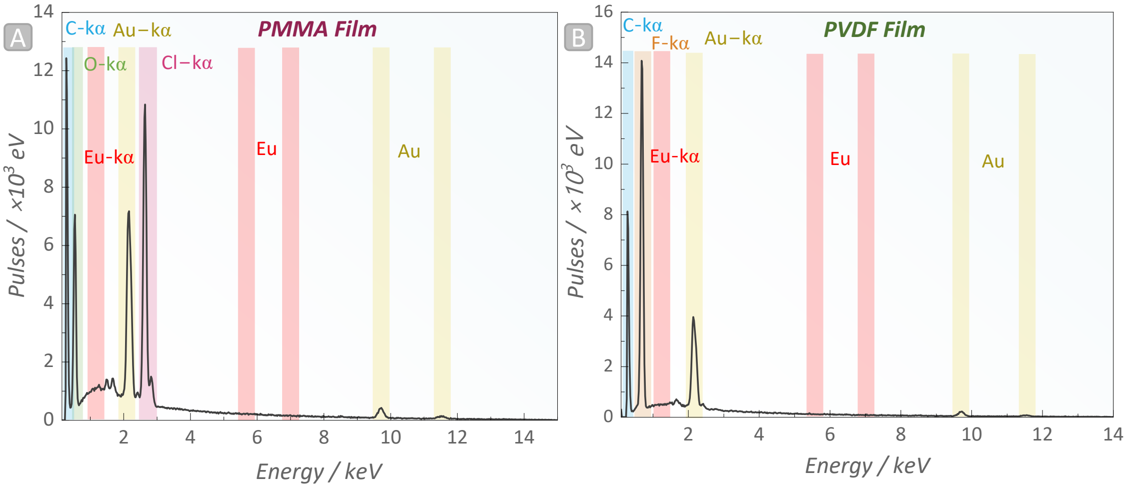

Fig S6. Energy dispersive spectroscopy of the (a) PMMA film and (b) PVDF film highlighting the observed peaks and their assignments. The films were prepared at 1 wt.% of Eu-complex with the same procedure documented in reference [19].

Energy dispersive x-ray spectra of the films (**Fig S6**) display peaks corresponding to Eu^{III}, although their low intensity reflects the low concentration of complex (1 wt.%). In the PMMA specimen, additional chlorine peaks appear, attributable to residual chloroform from the solvent used during film preparation. To verify the ionic distribution within the

technique detection limit, spatially resolved elemental maps were acquired for both films (**Fig S7** for PMMA and **Fig S8** for PVDF).

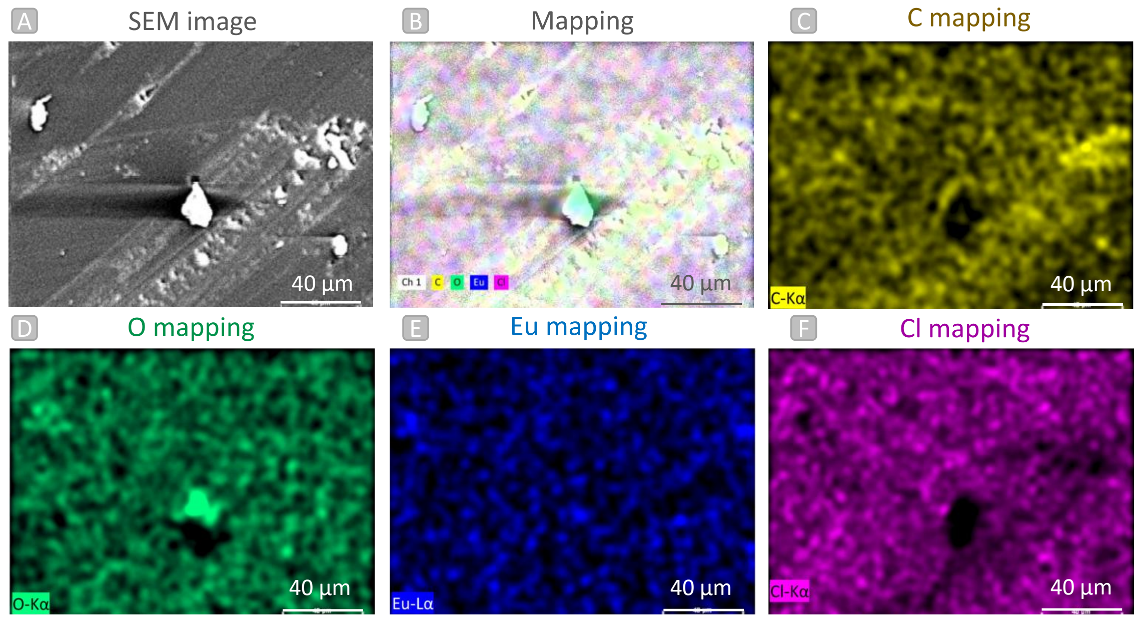

Fig S7. SEM image (A), chemical mapping of the 1 wt.% Eu-complex/PMMA film (B) and elemental maps (B-F) with a uniform element distribution of carbon (C), oxygen (D), europium (E), chlorine (F).

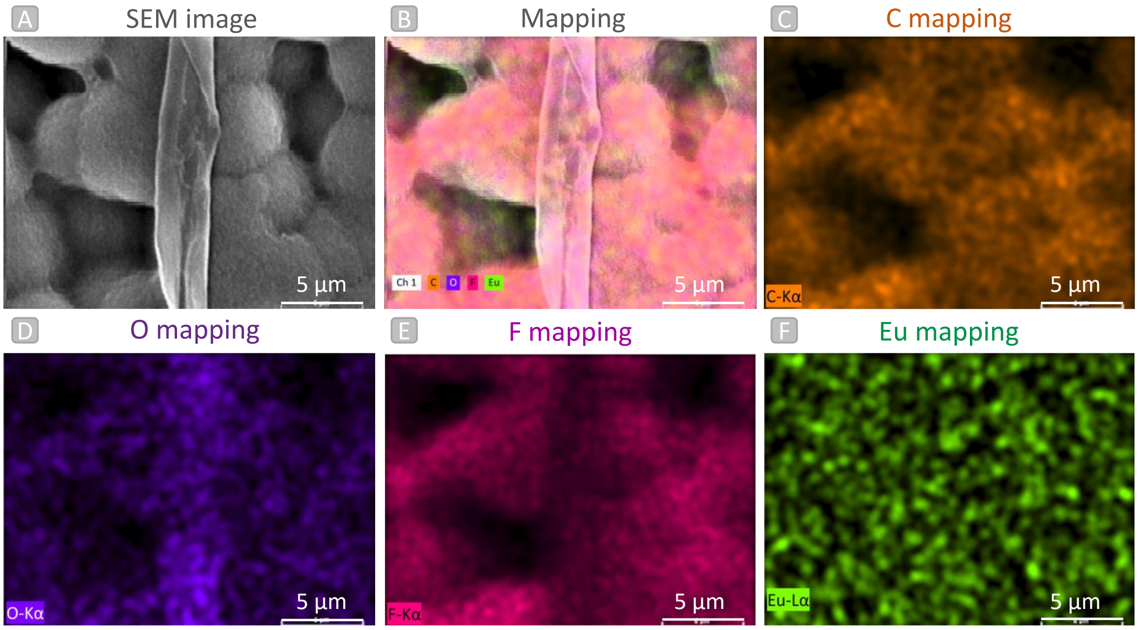

Fig S8. SEM image (A), chemical mapping of the 1 wt.% Eu-complex/PVDF film (B) and elemental maps (B-F) with a uniform element distribution of carbon (C), oxygen (D), fluorine (E), europium (F).

Supplementary Note S4 – Judd-Ofelt intensity parameters

Using the methodology presented in the Supplementary Note S1, we were able to compute the Judd-Ofelt intensity parameters, whose results are summarized in **Table S3 – S16.** The distortion in the axial ($\Delta\theta$) and equatorial ($\Delta\phi$) directions were obtained with a stepsize of 1.5°, i.e., if $\Delta\theta$ = 6°, the overall distortion is 8° due to the stepsize variation.

The distortion occurred as shown in **Fig S5 – S6**. Since these parameters are dependent on the polarizability of the environment around Eu^{III}, the calculation of the bond overlap polarizability was crucial, and the results are represented in **Fig S7.**

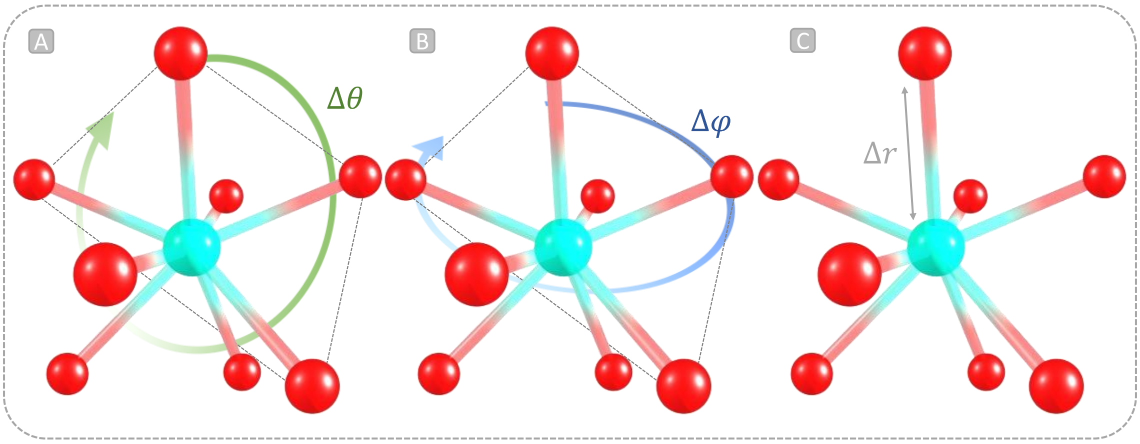

Fig S9. Plane of oxygens for the coordination polyhedron in the PMMA environment used in the distortion of angles (a) θ in the axial direction, (b) φ in the equatorial direction and (c) displacement of Eu−O bonds.

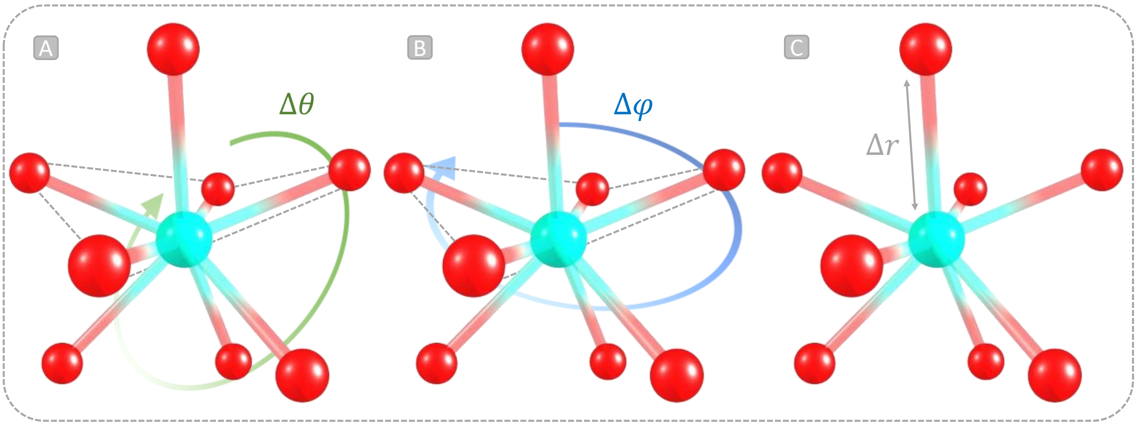

Fig S10. Plane of oxygens for the coordination polyhedron in the PVDF environment used in the distortion of angles (a) θ in the axial direction, (b) φ in the equatorial direction and (c) displacement of Eu−O bonds.

Table S3. Experimental and theoretical Judd-Ofelt intensity parameter Ω_2, obtained by distorting the Eu^{III} coordination polyhedron in the angle $\boldsymbol{\theta}$ of spherical coordinates (axial angle). The values are given in 10^{-20} cm^2 and are related to $[Eu(dbm)_4]^-$/PMMA. $\boldsymbol{\Delta\theta}$ is the degree angular variation in the axial axis, which was varied with a stepszie of 1.5°. The plane of oxygens in which the angles were varied is shown in Fig S5a. All experimental values were taken from reference [19].

Concentration / %	Intensity parameters		Distortion
	Ω_2^{exp}	Ω_2^{theo}	$\Delta\theta$ / °
**Complex**	19.0	18.9	0
**0.10**	–	–	–
**0.25**	17.0	17.1	−3
**0.50**	15.0	15.1	−8
**0.75**	23.0	23.4	6
**1.00**	25.0	25.3	8
**2.00**	26.0	26.1	9

Table S4. Experimental and theoretical Judd-Ofelt intensity parameter Ω_4, obtained by distorting the Eu^{III} coordination polyhedron in the angle $\boldsymbol{\theta}$ of spherical coordinates. The values are given in 10^{-20} cm^2 and are related to $[Eu(dbm)_4]^-$/PMMA. $\boldsymbol{\Delta\theta}$ is the degree angular variation in the axial axis, which was varied with a stepszie of 1.5°. The plane of oxygens in which the angles were varied is shown in Fig S5a. All experimental values were taken from reference [19].

Concentration / %	Intensity parameters		Distortion
	Ω_4^{exp}	Ω_4^{theo}	$\Delta\theta$ / °
**Complex**	1.20	1.23	0
**0.1**	–	–	–
**0.25**	1.10	1.09	5
**0.5**	1.20	1.20	1
**0.75**	1.50	1.49	−10
**1.0**	1.80	–	–
**2.0**	1.20	1.20	1

Table S5. Experimental and theoretical Judd-Ofelt intensity parameters Ω_2, obtained by distorting the Eu[III] coordination polyhedron in the angle $\boldsymbol{\varphi}$ (equatorial angle) of spherical coordinates. The values are given in 10^{-20} cm^2 and are related to $[Eu(dbm)_4]^-$/PMMA. $\boldsymbol{\Delta\varphi}$ is the degree angular variation in the equatorial axis, which was varied with a stepszie of 1.5°. The plane of oxygens in which the angles were varied is shown in Fig S5b. All experimental values were taken from reference [19].

Concentration / %	Intensity parameters		Distortion
	Ω_2^{exp}	Ω_2^{theo}	Δφ / °
**Complex**	19.0	18.9	0
**0.1**	–	–	–
**0.25**	17.0	17.1	−3
**0.5**	15.0	15.3	−6
**0.75**	23.0	22.9	6
**1.0**	25.0	25.2	10
**2.0**	26.0	26.2	12

Table S6. Experimental and theoretical Judd-Ofelt intensity parameter Ω_4, obtained by distorting the Eu[III] coordination polyhedron in the angle φ of spherical coordinates. The values are given in 10^{-20} cm^2 and are related to $[Eu(dbm)_4]^-$/PMMA. $\boldsymbol{\Delta\varphi}$ is the degree angular variation in the equatorial axis, which was varied with a stepszie of 1.5°. The plane of oxygens in which the angles were varied is shown in Fig S5b. All experimental values were take from reference [19].

Concentration / %	Intensity parameters		Distortion
	Ω_4^{exp}	Ω_4^{theo}	Δφ / °
**Complex**	1.20	1.23	0
**0.1**	–	–	–
**0.25**	1.10	1.09	3
**0.5**	1.20	1.28	−1
**0.75**	1.50	1.49	−11
**1.0**	1.80	–	–
**2.0**	1.20	1.28	−1

Table S7. Experimental and theoretical Judd-Ofelt intensity parameter Ω_2, obtained by distorting the Eu^{III} coordination polyhedron in the Eu−O bond length in forty steps of 0.015 Å. The values are given in 10^{-20} cm^2 and are related to $[Eu(dbm)_4]^-$/PMMA. The oxygen atoms which was displeced is shown in Fig S5c. All experimental values were taken from reference [19].

Concentration / %	Intensity parameters		Displacement
	Ω_2^{exp}	Ω_2^{theo}	Δr / Å
**Complex**	19.0	18.9	0
**0.1**	–	–	–
**0.25**	17.0	17.2	0.04
**0.5**	15.0	15.2	0.09
**0.75**	23.0	23.1	−0.07
**1.0**	25.0	25.2	−0.10
**2.0**	26.0	25.9	−0.11

Table S8. Experimental and theoretical Judd-Ofelt intensity parameter Ω_4, obtained by distorting the Eu^{III} coordination polyhedron in the Eu−O bond length in forty steps of 0.015 Å. The values are given in 10^{-20} cm^2 and are related to $[Eu(dbm)_4]^-$/PMMA. The oxygen atoms which was displeced is shown in Fig S5c. All experimental values were taken from reference [19].

Concentration / %	Intensity parameters		Displacement
	Ω_4^{exp}	Ω_4^{theo}	Δr / Å
**Complex**	1.20	18.9	0
**0.1**	–	–	–
**0.25**	1.10	1.10	0.09
**0.5**	1.20	1.20	0.02
**0.75**	1.50	1.49	−0.11
**1.0**	1.80	1.82	−0.20
**2.0**	1.20	1.20	0.02

Table S9. Derived factors for the calculation of the Judd-Ofelt intensity parameters. ρ_j represents the overlap integral between Eu−O, α_{OP} is the bond overlap polarizability given in 10^{-26} cm^3, g_j is the dimensionless charge factor, and α_j' is the effective polarizability induced by the ligands on Eu^{III} surroundings in Å^{-3}.

Bond	Length / Å	ρ_j / e	α_{OP}	g_j	α_j'
**Eu−O(1)**	2.530	0.0532	2.906	0.0590	1.437
**Eu−O(2)**	2.533	0.0529	2.890	0.0870	0.0530
**Eu−O(3)**	2.529	0.0532	2.910	0.0590	1.437
**Eu−O(4)**	2.531	0.0530	2.898	0.0870	0.0530
**Eu−O(5)**	2.529	0.0532	2.911	0.0590	1.437
**Eu−O(6)**	2.521	0.0530	2.897	0.0870	0.0530
**Eu−O(7)**	2.529	0.0532	2.911	0.0590	1.437
**Eu−O(8)**	2.532	0.0523	2.894	0.0870	0.0530

Table S10. Calculated relative (%) contributions of the forced electric dipole (FED) alongside the dynamics coupling (DC) mechanism to the total theoretical intensity parameters, and the contribution of the bond overlap polarizability to the intensity parameters of the isolated complex.

Compound	FED / %			DC / %			α_{OP} / %		
	Ω_2	Ω_4	Ω_6	Ω_2	Ω_4	Ω_6	Ω_2	Ω_4	Ω_6
$[Eu(dbm)_4]^-$	0.01	1.2	3.8	99.9	98.8	96.2	1.02	2.31	8.23

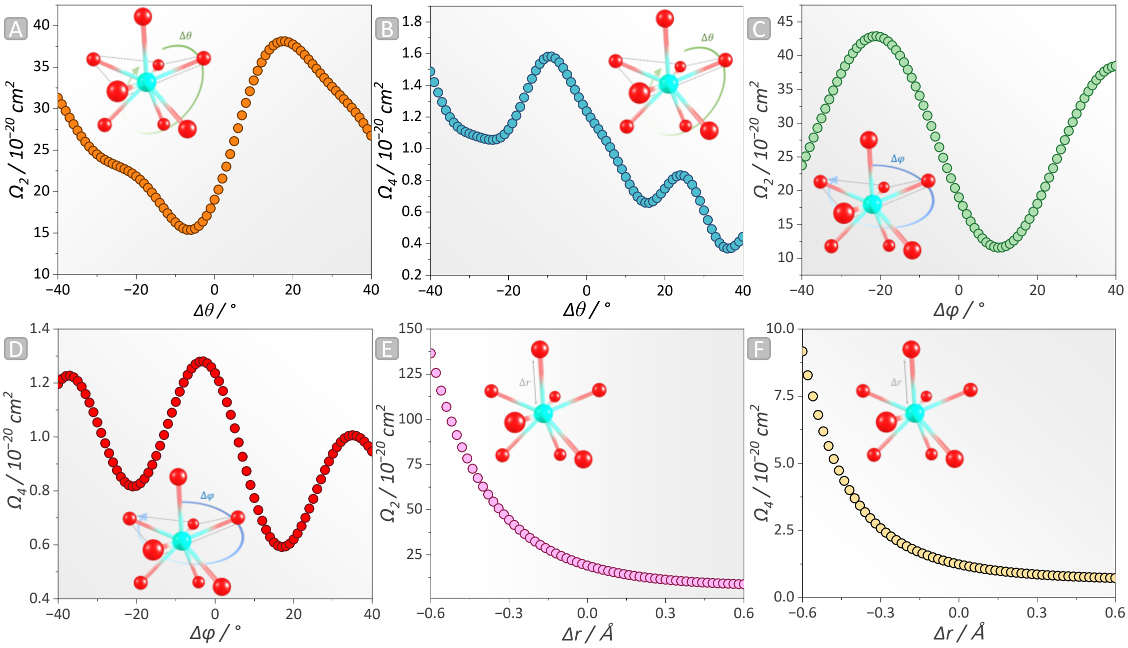

Fig S11. Trends in the theoretical Judd-Ofelt intensity parameters in $[Eu(dbm)_4]^-$/PVDF by varying the θ angle in (a) Ω_2 and (b) Ω_4, φ angle in (c) Ω_2 and (d) Ω_4, followed by variations in the Eu–O distances in (e) Ω_2 and (f) Ω_4.

Table S11. Experimental and theoretical Judd-Ofelt intensity parameters (Ω_2) obtaining by distorting the Eu^{III} coordination polyhedron in the angle $\boldsymbol{\theta}$ (axial axis) of spherical coordinates. The values are given in 10^{-20} cm^2 and are related to $[Eu(dbm)_4]^-$/PVDF. $\boldsymbol{\Delta\theta}$ is the degree angular variation in the axial axis, which was varied with a stepszie of 1.5°. The plane of oxygens in which the angles were varied is shown in Fig S6a. All experimental values were taken from reference [19].

Concentration / %	Intensity parameters		Distortion
	Ω_2^{exp}	Ω_2^{theo}	$\Delta\theta$ / °
**Complex**	19.0	18.9	0
**0.10**	–	–	–
**0.25**	–	–	–
**0.50**	32.0	32.1	9
**0.75**	33.0	33.3	10
**1.00**	30.0	30.2	8
**2.00**	35.0	35.4	12

Table S12. Experimental and theoretical Judd-Ofelt intensity parameters (Ω_4) obtaining by distorting the Eu^{III} coordination polyhedron in the angle $\boldsymbol{\theta}$ of spherical coordinates. The values are given in 10^{-20} cm^2 and are related to [Eu(dbm)$_4$]$^-$/PVDF. $\boldsymbol{\Delta\theta}$ is the degree angular variation in the axial axis, which was varied with a stepszie of 1.5°. The plane of oxygens in which the angles were varied is shown in Fig S6a. All experimental values were taken from reference [19].

Concentration / %	Intensity parameters		Distortion
	Ω_4^{exp}	Ω_4^{theo}	$\Delta\theta$ / °
**Complex**	1.20	1.23	0
**0.10**	–	–	–
**0.25**	–	–	–
**0.50**	6.50	–	–
**0.75**	6.30	–	–
**1.0**	5.70	–	–
**2.0**	5.90	–	–

Table S13. Experimental and theoretical Judd-Ofelt intensity parameters (Ω_2) obtaining by distorting the Eu^{III} coordination polyhedron in the angle $\boldsymbol{\varphi}$ (equatorial axis) of spherical coordinates. The values are given in 10^{-20} cm^2 and are related to [Eu(dbm)$_4$]$^-$/PVDF. $\boldsymbol{\Delta\varphi}$ is the degree angular variation in the axial axis, which was varied with a stepszie of 1.5°. The plane of oxygens in which the angles were varied is shown in Fig S6b. All experimental values were taken from reference [19].

Concentration / %	Intensity parameters		Distortion
	Ω_2^{exp}	Ω_2^{theo}	$\Delta\varphi$ / °
**Complex**	19.0	18.9	0
**0.10**	–	–	–
**0.25**	–	–	–
**0.50**	32.0	32.6	−9
**0.75**	33.0	33.3	−10
**1.0**	30.0	29.8	−7
**2.0**	35.0	35.4	−11

Table S14. Experimental and theoretical Judd-Ofelt intensity parameters (Ω_4) obtaining by distorting the Eu^{III} coordination polyhedron in the angle $\boldsymbol{\varphi}$ (equatorial axis) of spherical coordinates. The values are given in 10^{-20} cm^2 and are related to $[Eu(dbm)_4]^-$/PVDF. $\boldsymbol{\Delta\varphi}$ is the degree angular variation in the axial axis, which was varied with a stepszie of 1.5°. The plane of oxygens in which the angles were varied is shown in Fig S6b. All experimental values were taken from reference [19].

Concentration / %	Intensity parameters		Distortion
	Ω_4^{exp}	Ω_4^{theo}	$\Delta\varphi$ / °
**Complex**	1.20	1.23	0
**0.10**	–	–	–
**0.25**	–	–	–
**0.50**	6.50	–	–
**0.75**	6.30	–	–
**1.0**	5.70	–	–
**2.0**	5.90	–	–

Table S15. Experimental and theoretical Judd-Ofelt intensity parameters (Ω_2) obtaining by distorting the Eu^{III} coordination polyhedron in the Eu−O bond length in forty steps of 0.015 Å. The values are given in 10^{-20} cm^2 and are related to $[Eu(dbm)_4]^-$/PVDF. The oxygen atoms which was displeced is shown in Fig S6c. All experimental values were taken from reference [19].

Concentration / %	Intensity parameters		Distortion
	Ω_2^{exp}	Ω_2^{theo}	Δr / Å
**Complex**	19.0	18.9	0
**0.10**	–	–	–
**0.25**	–	–	–
**0.50**	32.0	32.3	−0.203
**0.75**	33.0	34.0	−0.223
**1.0**	30.0	30.4	−0.182
**2.0**	35.0	35.5	−0.241

Table S16. Experimental and theoretical Judd-Ofelt intensity parameters (Ω_4) obtaining by distorting the Eu^{III} coordination polyhedron in the Eu–O bond length in forty steps of 0.015 Å. The values are given in 10^{-20} cm^2 and are related to $[Eu(dbm)_4]^-$/PVDF. The oxygen atoms which was displeced is shown in Fig S6c. All experimental values were taken from reference [19].

Concentration / %	Intensity parameters		Distortion
	Ω_4^{exp}	Ω_4^{theo}	Δr / Å
**Complex**	1.20	18.9	0
**0.10**	–	–	–
**0.25**	–	–	–
**0.50**	6.50	6.51	−0.531
**0.75**	6.30	6.29	−0.524
**1.0**	5.70	5.69	−0.503
**2.0**	5.90	5.90	−0.511

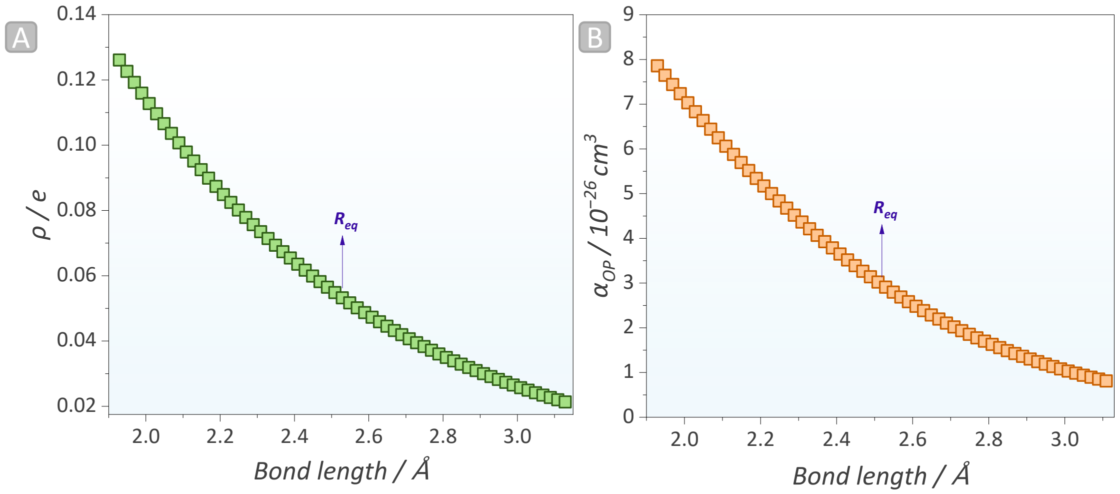

Fig S12. Trends in (a) overlap integral and (b) bond overlap polarizability, $\boldsymbol{\alpha_{OP}}$ as a function of the absolute variation in the bond length.

Supplementary note S5 – Electronic structure

Table S17. Percentage contribution of the molecular orbitals to the formation of S_1 and T_1 states of the complex in PMMA. Only the MOs with contribution greater than 5% were selected. L = LUMO. These molecular orbitals are graphically pictured in **Fig S7.**

Complex	S_1 state	T_1 state
**$[Eu(dbm)_4]^-$**	29% L+3; 22% L+2; 14% L+3	59% L+2; 12% L+3; 9% L+4

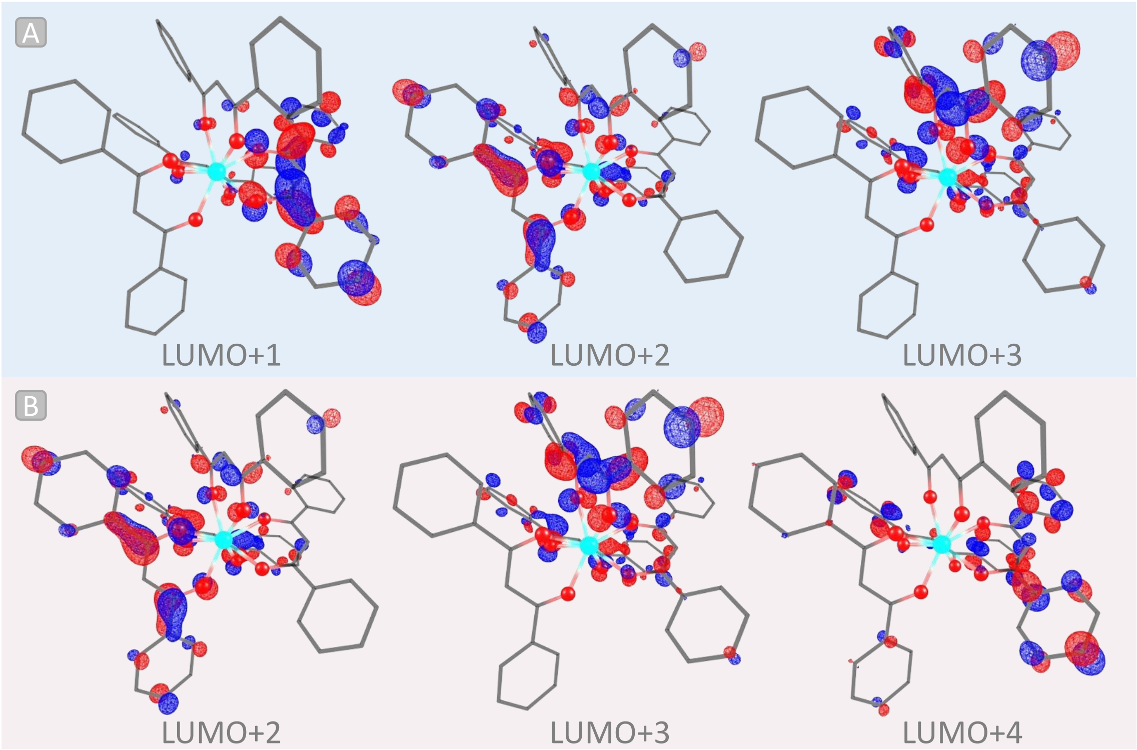

Fig S13. Most contributing molecular orbitals to the (a) S_1 and (b) T_1 states of the $[Eu(dbm)_4]^-$ complex in PMMA. Cyan = europium, gray = carbon, red = oxygen. The hydrogen atoms were hidden for clarity.

Table S18. Percentage contribution of the molecular orbitals to the formation of S_1 and T_1 states of the complex in PVDF. Only the MOs with contribution greater than 5% were selected. L = LUMO. These molecular orbitals are graphically pictured in **Fig S8.**

Complex	S_1 state	T_1 state
**$[Eu(dbm)_4]^-$**	24% L+3; 21% L; 17% L+1	72% L+1; 6% L+2; 5% L+3

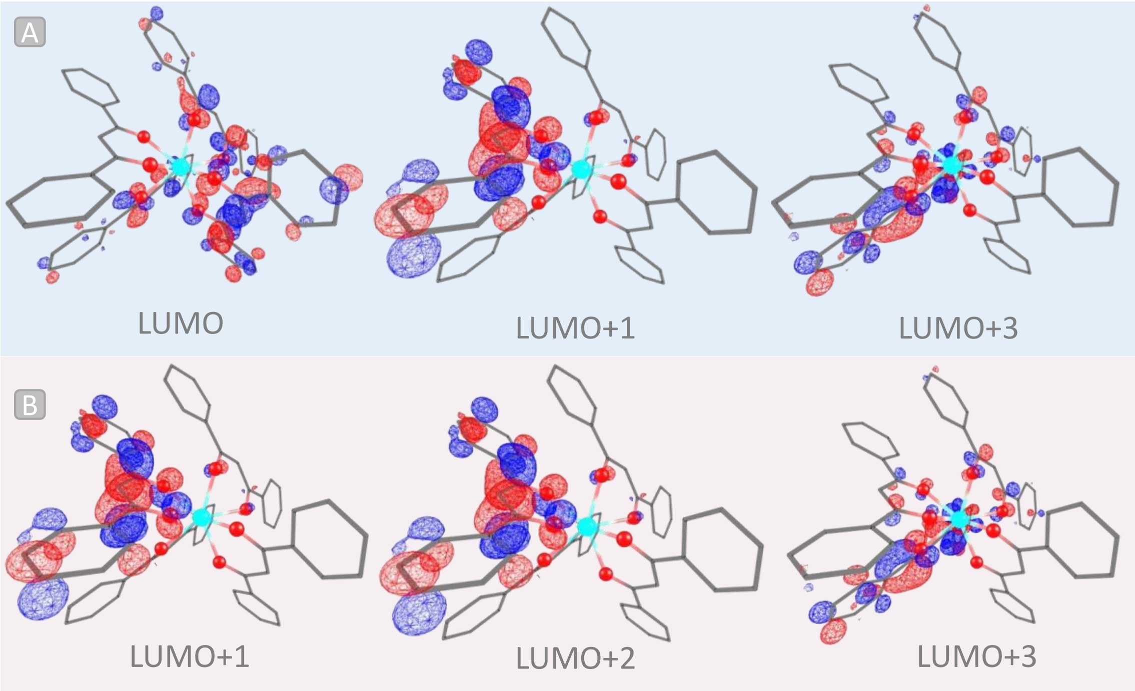

Fig S14. Most contributing molecular orbitals to the (a) S_1 and (b) T_1 states for the $[Eu(dbm)_4]^-$ complex in PVDF. Cyan = europium, gray = carbon, red = oxygen. The hydrogen atoms were hidden for clarity.

Table S19. Energies of singlet and triplet state and their donor-acceptor distance (R_L). All values were obtained according to the molecular orbital analysis.

$[Eu(dbm)_4]^-$	Singlet (S_1)		Triplet (T_1)	
	R_L / Å	Energy / cm^{-1}	R_L / Å	Energy / cm^{-1}
**PMMA**	4.688	34248	4.828	21791
**PVDF**	4.826	34211	4.808	22790

Supplementary note S6 – Energy transfer and populations

Table S20. Energy transfer rates (in s^{-1}) for $[Eu(dbm)_4]^-$ complex in PMMA considering only the states that contribute more than 0.001% for S_1 and T_1. δ is the donor-acceptor (singlet/triplet-Eu^{3+}) energy difference (in cm^{-1}). W_{d-d}, W_{m-d}, W_{ex} are the dipole-dipole, dipole-multipole, and exchange rates (in cm^{-1}), respectively. $\boldsymbol{W}$ and $\boldsymbol{W^b}$ are the forward and back-energy transfer rates (s^{-1}), respectively, at 298.15 K.

Pathway	Donor	Accept	δ	W_{d-d}	W_{m-d}	W_{ex}	W	W^b
**10**	S_1	$^7F_0 \rightarrow {^5L_6}$	8923	3.113×10^{0}	2.593×10^{1}	0	2.905×10^{1}	5.347×10^{-19}
**15**	S_1	$^7F_0 \rightarrow {^5G_6}$	7496	4.575×10^{0}	3.811×10^{1}	0	4.268×10^{1}	7.689×10^{-16}
**18**	S_1	$^7F_0 \rightarrow {^5D_4}$	6662	1.828×10^{0}	4.421×10^{3}	0	4.423×10^{3}	6.439×10^{-12}
**29**	S_1	$^7F_0 \rightarrow {^5D_4}$	10265	1.400×10^{-3}	2.879×10^{2}	0	2.879×10^{2}	2.283×10^{-19}
**31**	S_1	$^7F_1 \rightarrow {^5L_7}$	8263	5.858×10^{-1}	4.880×10^{0}	0	5.466×10^{0}	3.173×10^{-17}
**32**	S_1	$^7F_1 \rightarrow {^5G_2}$	8228	0	0	1.306×10^{5}	1.306×10^{5}	2.693×10^{-12}
**33**	S_1	$^7F_1 \rightarrow {^5G_3}$	7998	3.163×10^{-2}	3.527×10^{3}	0	3.527×10^{3}	1.576×10^{-13}
**35**	S_1	$^7F_1 \rightarrow {^5G_6}$	7868	2.591×10^{-1}	2.158×10^{0}	0	2.417×10^{0}	1.089×10^{-16}
**36**	S_1	$^7F_1 \rightarrow {^5G_5}$	7857	5.313×10^{-1}	3.238×10^{1}	0	3.292×10^{1}	1.849×10^{-15}
**Total**	S_1						1.390×10^{5}	9.292×10^{-12}
**7**	T_1	$^7F_0 \rightarrow {^5D_1}$	2764	0	0	1.031×10^{8}	1.031×10^{-8}	6.647×10^{1}
**26**	T_1	$^7F_1 \rightarrow {^5D_0}$	4870	0	0	8.508×10^{6}	8.508×10^{6}	9.561×10^{-3}
**28**	T_1	$^7F_1 \rightarrow {^5D_2}$	680	0	0	1.997×10^{6}	1.997×10^{6}	2.710×10^{5}
**32**	T_1	$^7F_1 \rightarrow {^5G_2}$	−4229	0	0	2.001×10^{3}	2.001×10^{3}	5.268×10^{6}
**Total**	T_1						1.136×10^{8}	5.540×10^{6}

Table S21. Energy transfer rates (in s^{-1}) for $[Eu(dbm)_4]^-$ complex in PVDF considering only the states that contribute more than 0.001% for S_1 and T_1. δ is the donor-acceptor (singlet/triplet-Eu^{3+}) energy difference (in cm^{-1}). W_{d-d}, W_{m-d}, W_{ex} are the dipole-dipole, dipole-multipole, and exchange rates (in cm^{-1}), respectively. $\boldsymbol{W}$ and $\boldsymbol{W^b}$ are the forward and back-energy transfer rates (s^{-1}), respectively, at 298.15 K.

Pathway	Donor	Accept	δ	W_{d-d}	W_{m-d}	W_{ex}	W	W^b
**10**	S_1	$^7F_0 \rightarrow ^5L_6$	8886	2.949×10^{1}	1.834×10^{2}	0	2.129×10^{2}	4.686×10^{-18}
**15**	S_1	$^7F_0 \rightarrow ^5G_6$	7459	1.959×10^{1}	1.219×10^{2}	0	1.414×10^{2}	3.046×10^{-15}
**18**	S_1	$^7F_0 \rightarrow ^5D_4$	6625	5.243×10^{0}	1.064×10^{4}	0	1.065×10^{4}	1.853×10^{-11}
**29**	S_1	$^7F_0 \rightarrow ^5D_4$	10228	3.172×10^{-2}	6.146×10^{3}	0	6.146×10^{3}	5.827×10^{-18}
**31**	S_1	$^7F_1 \rightarrow ^5L_7$	8226	3.779×10^{0}	2.350×10^{1}	0	2.728×10^{1}	1.894×10^{-16}
**32**	S_1	$^7F_1 \rightarrow ^5G_2$	8191	0	0	7.138×10^{5}	7.138×10^{5}	1.760×10^{-11}
**33**	S_1	$^7F_1 \rightarrow ^5G_3$	7961	1.763×10^{-1}	1.850×10^{4}	0	1.850×10^{4}	9.886×10^{-13}
**35**	S_1	$^7F_1 \rightarrow ^5G_6$	7831	1.346×10^{0}	8.375×10^{0}	0	9.722×10^{0}	5.237×10^{-16}
**36**	S_1	$^7F_1 \rightarrow ^5G_5$	7820	2.745×10^{0}	1.384×10^{2}	0	1.411×10^{2}	9.474×10^{-15}
**Total**	S_1						7.721×10^{5}	3.713×10^{-11}
**7**	T_1	$^7F_0 \rightarrow ^5D_1$	3763	0	0	7.890×10^{7}	7.890×10^{7}	4.100×10^{-1}
**26**	T_1	$^7F_1 \rightarrow ^5D_0$	5869	0	0	9.325×10^{6}	9.325×10^{6}	8.447×10^{-5}
**28**	T_1	$^7F_1 \rightarrow ^5D_2$	1679	0	0	1.437×10^{6}	1.437×10^{6}	1.572×10^{3}
**32**	T_1	$^7F_1 \rightarrow ^5G_2$	−3230	0	0	4.913×10^{-1}	4.913×10^{-1}	1.043×10^{7}
**Total**	T_1						8.967×10^{7}	1.043×10^{7}

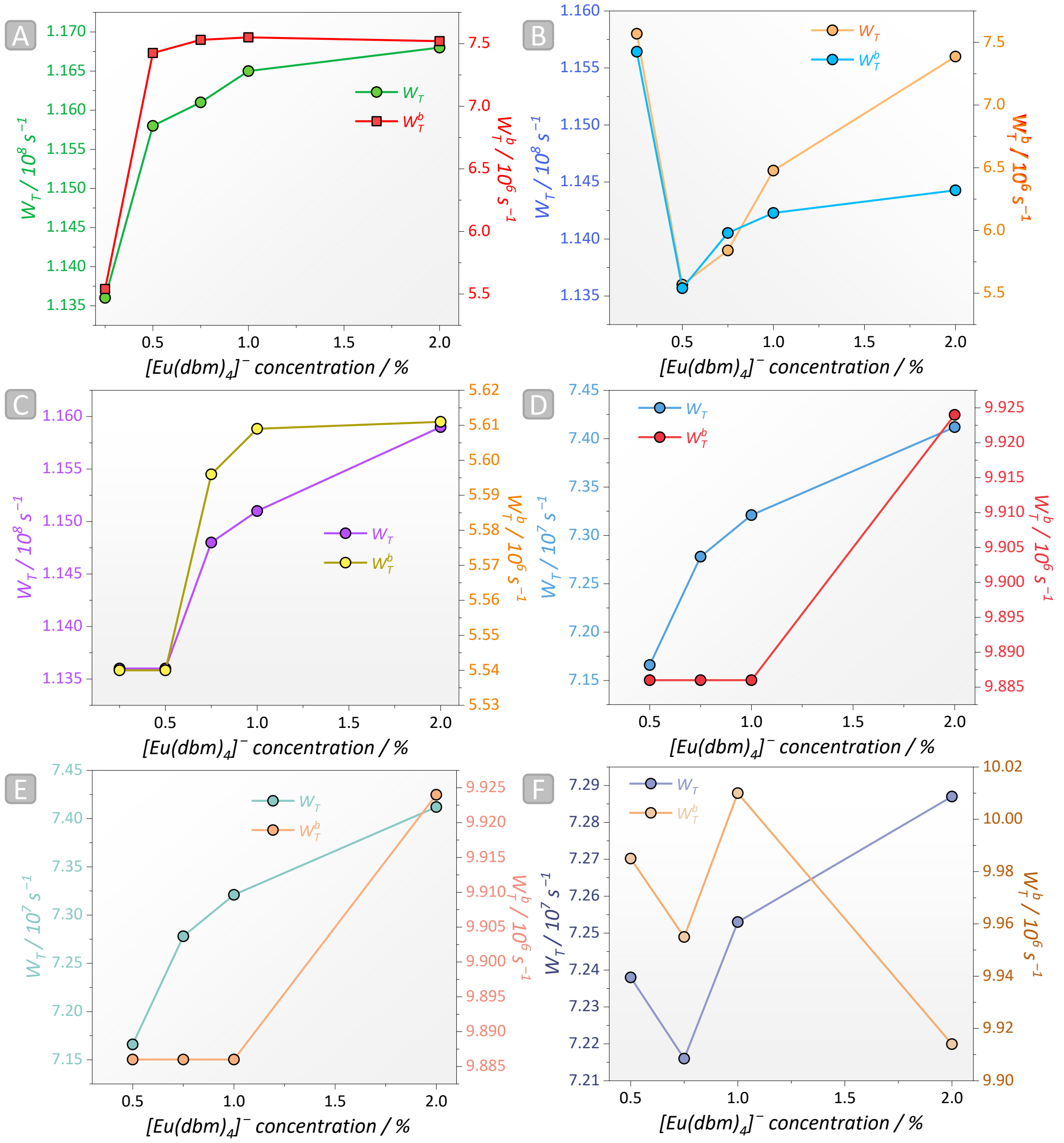

Fig S15. Rates of energy transfer by varying the coordination polyhedron for [Eu(dbm)4]⁻/PMMA modifying (a) $\Delta\theta$; (b) $\Delta\varphi$; (c) Δr and [Eu(dbm)4]⁻/PVDF modifying (d) $\Delta\theta$; (e) $\Delta\varphi$; (f) Δr.

Table S22. Total rates of forward and backward energy transfer from the singlet state (W_S and W_S^b, respectively) for the [Eu(dbm)4]⁻/PMMA film.

[Eu(dbm)4]⁻/PMMA	W_S / s^{-1}			W_S^b/ s^{-1}		
	$\Delta\theta$	$\Delta\varphi$	Δr	$\Delta\theta$	$\Delta\varphi$	Δr
**0.25**	1.390×10^5	1.390×10^5	1.390×10^5	9.292×10^{-12}	9.293×10^{-12}	9.293×10^{-12}
**0.50**	1.064×10^6	1.390×10^5	1.390×10^5	4.235×10^{-11}	9.293×10^{-12}	9.293×10^{-12}
**0.75**	1.034×10^6	1.420×10^5	1.413×10^5	4.235×10^{-11}	9.312×10^{-12}	9.364×10^{-12}
**1.00**	1.034×10^6	1.430×10^5	1.422×10^5	4.235×10^{-11}	9.334×10^{-12}	9.392×10^{-12}
**2.00**	1.039×10^6	1.430×10^5	1.424×10^5	4.239×10^{-11}	9.334×10^{-12}	9.401×10^{-12}

Table S23. Total rates (in s^{-1}) of forward and backward energy transfer from the singlet state (W_S and W_S^b, respectively) for the $[Eu(dbm)_4]^-$/PVDF film.

$[Eu(dbm)_4]^-$/PVDF	W_S / s^{-1}			W_S^b/ s^{-1}		
	$\Delta\theta$	$\Delta\varphi$	Δr	$\Delta\theta$	$\Delta\varphi$	Δr
**0.50**	1.057×10^5	1.057×10^5	1.099×10^5	8.238×10^{-12}	8.237×10^{-12}	8.425×10^{-12}
**0.75**	1.121×10^5	1.121×10^5	1.102×10^5	8.241×10^{-12}	8.241×10^{-12}	8.448×10^{-12}
**1.00**	1.057×10^5	1.057×10^5	1.096×10^5	8.239×10^{-12}	8.239×10^{-12}	8.402×10^{-12}
**2.00**	1.141×10^5	1.141×10^5	1.105×10^5	8.240×10^{-12}	8.240×10^{-12}	8.473×10^{-12}

Table S24. Total rates of forward and backward energy transfer from the triplet state (W_T and W_T^b, respectively) for the $[Eu(dbm)_4]^-$/PMMA film.

$[Eu(dbm)_4]^-$/PMMA	W_T / s^{-1}			W_T^b/ s^{-1}		
	$\Delta\theta$	$\Delta\varphi$	Δr	$\Delta\theta$	$\Delta\varphi$	Δr
**0.25**	1.136×10^8	1.158×10^8	1.136×10^8	5.540×10^6	7.425×10^6	5.540×10^6
**0.50**	1.158×10^8	1.136×10^8	1.136×10^8	7.425×10^6	5.540×10^6	5.540×10^6
**0.75**	1.161×10^8	1.139×10^8	1.148×10^8	7.530×10^6	5.980×10^6	5.596×10^6
**1.00**	1.165×10^8	1.146×10^8	1.151×10^8	7.550×10^6	6.140×10^6	5.609×10^6
**2.00**	1.168×10^8	1.156×10^8	1.159×10^8	7.520×10^6	6.320×10^6	5.611×10^6

Table S25. Total rates of forward and backward energy transfer from the triplet state (W_T and W_T^b, respectively) for the $[Eu(dbm)_4]^-$/PVDF film.

$[Eu(dbm)_4]^-$/PVDF	W_T / s^{-1}			W_T^b/ s^{-1}		
	$\Delta\theta$	$\Delta\varphi$	Δr	$\Delta\theta$	$\Delta\varphi$	Δr
**0.50**	7.166×10^7	7.166×10^7	7.238×10^7	9.886×10^6	9.886×10^6	9.985×10^6
**0.75**	7.278×10^7	7.278×10^7	7.216×10^7	9.886×10^6	9.886×10^6	9.955×10^6
**1.00**	7.321×10^7	7.321×10^7	7.253×10^7	9.886×10^6	9.886×10^6	1.001×10^7
**2.00**	7.412×10^7	7.412×10^7	7.287×10^7	9.924×10^6	9.924×10^6	9.914×10^6

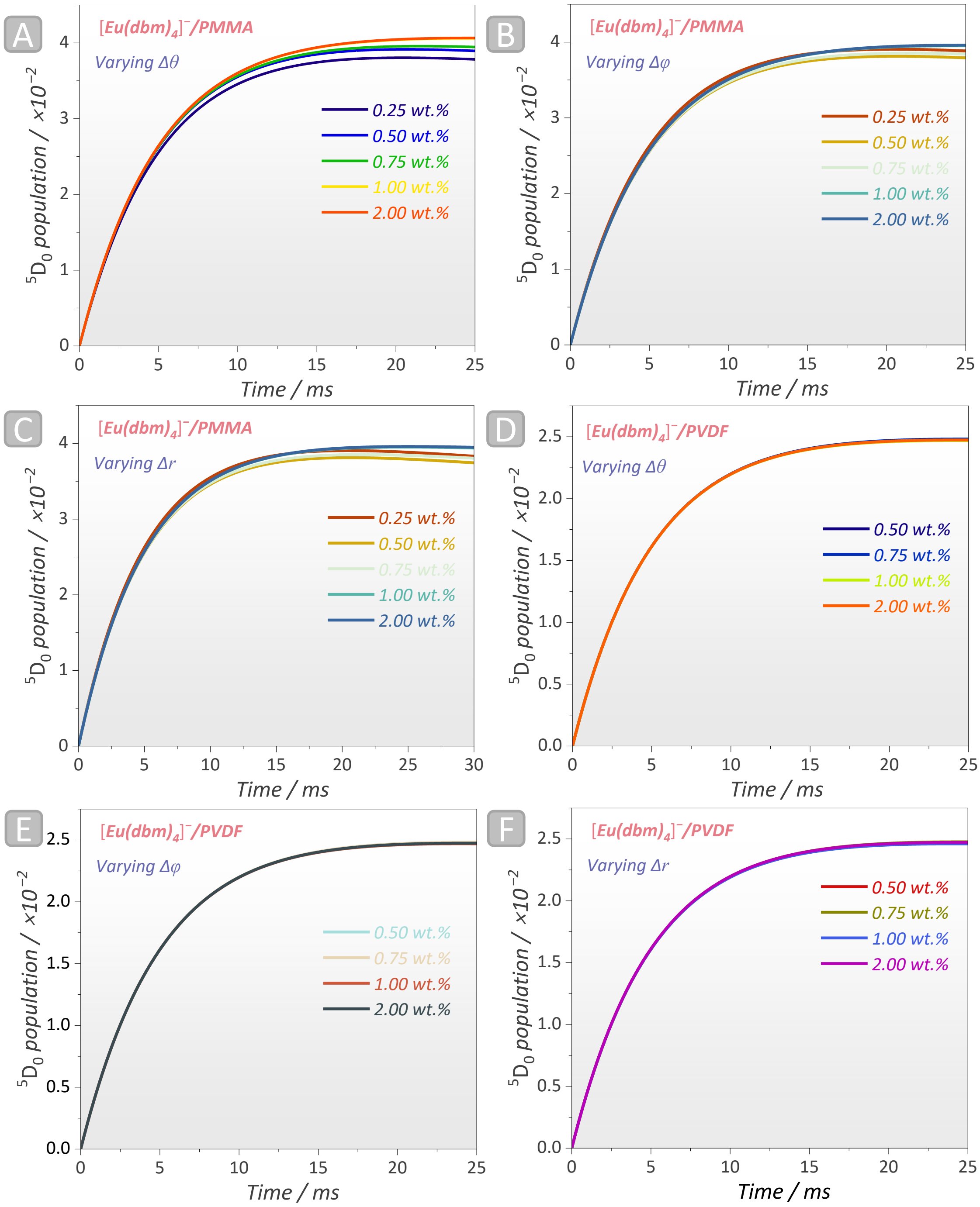

Fig S16. Population of the $^5D_0$ level obtained by the set of coupled ODEs by varying the coordination polyhedron for $[Eu(dbm)_4]^-$/PMMA modifying (a) $\Delta\theta$; (b) $\Delta\varphi$; (c) Δr and $[Eu(dbm)_4]^-$/PVDF modifying (d) $\Delta\theta$; (e) $\Delta\varphi$; (f) Δr.

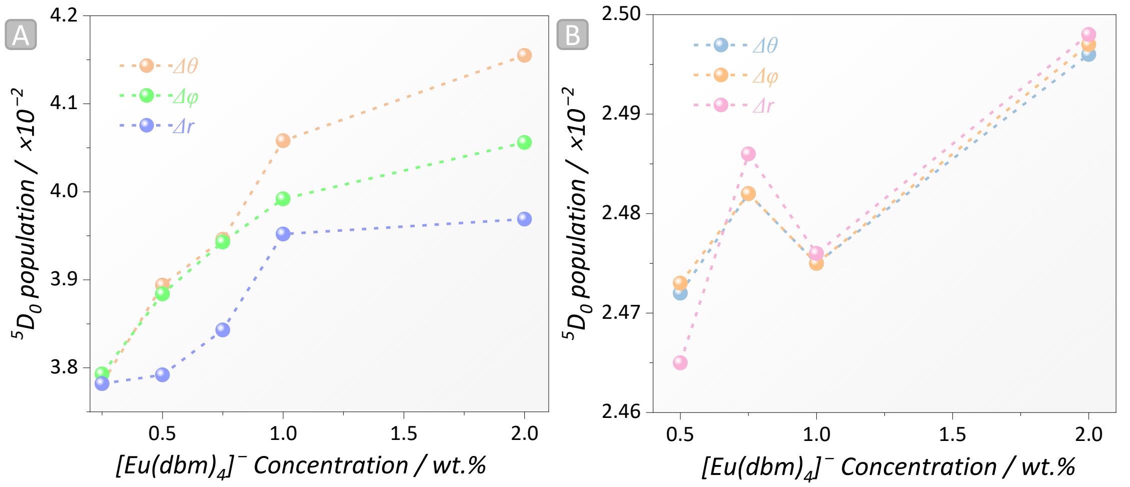

Fig S17. Population of $^5D_0$ manifold as a function of the concentration of the complex in (a) PMMA and (b) PVDF.

Table S26. Absolute population of $^5D_0$ state for the complex in PMMA and PVDF obtained after 25 ms. These populations were used to construct the graphs shown in **Fig S17**.

w.t.%	Population of the emissive state ($^5D_0$)					
	[Eu(dbm)$_4$]$^-$/PMMA			[Eu(dbm)$_4$]$^-$/PVDF		
	$\Delta\theta$	$\Delta\varphi$	Δr	$\Delta\theta$	$\Delta\varphi$	Δr
**0.25**	3.782×10^{-2}	3.793×10^{-2}	3.782×10^{-2}	–	–	–
**0.50**	3.894×10^{-2}	3.884×10^{-2}	3.792×10^{-2}	2.472×10^{-2}	2.473×10^{-2}	2.465×10^{-2}
**0.75**	3.946×10^{-2}	3.943×10^{-2}	3.843×10^{-2}	2.482×10^{-2}	2.482×10^{-2}	2.486×10^{-2}
**1.00**	4.058×10^{-2}	3.992×10^{-2}	3.952×10^{-2}	2.475×10^{-2}	2.475×10^{-2}	2.476×10^{-2}
**2.00**	4.155×10^{-2}	4.056×10^{-2}	3.969×10^{-2}	2.496×10^{-2}	2.497×10^{-2}	2.498×10^{-2}

To correctly estimate the values of theoretical quantum yield, it becomes necessary to calculate the theoretical radiative decay rates, which encompass both forced electric dipole and magnetic dipole contributions. In this sense, Eq. S20 and S21 report the employed expression, where *e* is the elementary charge, *n* the refractive index, ε_0 the vacuum permittivity, *h* the Planck constant, *c* the speed of light, $\sigma_{J'\rightarrow J}$ is the energetic barycenter of the $^5D_0 \rightarrow {}^7F_{0\text{-}4}$ band. In the same equations, Ω_λ denotes the theoretical Judd-Ofelt intensity parameters, SLJ and S'L'J' the ground and excited state manifold, and $\langle SLJ||U^{(\lambda)}||S'L'J'\rangle^2$ the squared reduced matrix elements with values of 0.0032 and 0.0023 for $\lambda = 2, 4$, respectively.

$$A_{ED} = \frac{32e^2\pi^3\left(\sigma_{J'\rightarrow J}\right)^3 n(n^2+2)^2}{27\hbar(2J+1)} \sum_{\lambda=2,4,6} \Omega_\lambda \left|\langle S'L'J'\|U^{(\lambda)}\|SLJ\rangle\right|^2 \quad \text{(S20)}$$

$$A_{MD} = \frac{32\pi^3\left(\sigma_{J'\rightarrow J}\right)^3 (\mu_B)^2 n^3}{3\hbar(2J+1)} |\langle S'L'J'\|L+gS\|SLJ\rangle|^2 \quad \text{(S21)}$$

$$A_{rad} = A_{ED} + A_{MD} \quad \text{(S22)}$$

References

[1] Judd, B. R. Optical Absorption Intensities of Rare-Earth Ions, *Phys. Rev.* 1962, 127, 750.
[2] Ofelt, G. S. Intensities of Crystal Spectra of Rare-Earth Ions, *J. Chem. Phys.* 1962, 37, 511.
[3] Malta, O. L. A simple overlap model in lanthanide crystal-field theory, *Chem. Phys. Lett.* 1982, 87, 27.
[4] Moura-Jr, R. T.; Carneiro Neto, A. N.; Longo, R. L.; Malta, O. L. On the calculation and interpretation of covalency in the intensity parameters of 4*f*-4*f* transitions in Eu^{3+} complexes based on the chemical bond overlap polarizability, J. *Lumin*. 2016, 170, 420 – 430.
[5] Trannoy, V.; Carneiro Neto, A. N.; Brites, C. D. S.; Carlos, L. D.; Serier-Brault, H. Engineering of Mixed Eu^{3+}/Tb^{3+} Metal-Organic Frameworks Luminescent Thermometers with Tunable Sensitivity, *Adv. Opt. Mat.*, 2021, 9, 200193.
[6] Malta, O. L.; Gouveia, E. A. Comment on the average energy denominator method in perturbation theory, *Phys. Lett. A* 1983, 97, 333.
[7] Moura Jr, R. T.; Neto, A. N. C.; Aguiar, E. C.; Santos Jr, C. V.; de Lima, E. M.; Faustino, W. M.; Teotonio, E. E. S.; Brito, H. F.; Felinto, M. C. F. C.; Ferreira, R. A. S.; Carlos, L. D.; Longo, R. L.; Malta, O. L. JOYSpectra: A web platform for luminescence of lanthanides, *Opt. Mat. X* 2021, 11, 100080.
[8] Santos-Jr., C. V.; Aguiar, E. C.; Carneiro Neto, A. N.; Moura Jr., R. T. Adaptive guided stochastic optimization: A novel approach for fitting the theoretical intensity parameters for lanthanide compounds, *Opt. Mat. X* 2023, 20, 100275.
[9] Moura Jr., R. T.; Quintano, M.; Santos-Jr., C. V.; Albuquerque, V. A. C. A.; Aguiar, E. C.; Kraka, E.; Carneiro Neto, A. N. Featuring a new computational protocol for the estimation of intensity and overall quantum yield in lanthanide chelates with applications to Eu(III) mercapto-triazole Schiff base Ligands, *Opt. Mat. X* 2022, 16, 100216.
[10] Carneiro Neto, A. N.; Moura Jr, R. T. Overlap integrals and excitation energies calculations in trivalent lanthanides 4*f* orbitals in pairs Ln-L (L =Ln, N, O, F, P, S, Cl, Se, Br, and I). *Chem. Phys. Lett.* 2020, 757, 137884.
[11] Carneiro Neto, A. N.; Teotonio, E. E. S.; de Sá, G. F.; Brito, H. F.; Legendziewicz, J.; Carlos, L. D.; Felinto, M. C. F. C.; Gawryszewska, P.; Moura Jr, R. T.; Longo, R. L.; Faustino, W. M.; Malta, O. L. *Chapter 310 – Modeling intramolecular energy transfer in lanthanide chelates. A critical review and recent advances*. *In: Handbook on the Physics and Chemistry of Rare Earths*, 2019, 56, pp. 55 – 162.
[12] Malta, O. L. Ligand-rare-earth ion energy transfer in coordination compounds. A theoretical approach, *J. Lumin.* 1997, 71, 229.
[13] Malta, O. L.; Gonçalves e Silva, F. R. A theoretical approach to intramolecular energy transfer and emission quantum yields in coordination compounds of rare earth ions, *Spectrochim. Acta A* 1998, 54, 1593.
[14] Malta, O. L. Mechanisms of non-radiative energy transfer involving lanthanide ions revisited, *J. Non-Cryst. Sol.* 2008, 354, 4770.
[15] Carnall, W. T.; Fields, P. R.; Rajnak, K. Electronic Energy Levels of the Trivalent Lanthanide Aquo Ions. IV. Eu^{3+}, *J. Chem. Phys.* 1968, 49, 4450.
[16] Ofelt, G. S. Structure of the f^6 Configuration with Application to Rare Earth-Ions, *J. Chem. Phys.* 1963, 38, 2171.
[17] Kasprzycka, E.; Carneiro Neto, A. N.; Trush, V. A.; Jerzykiewicz, L.; Amirkhanov, V. M.; Malta, O. L.; Legendziewicz, J.; Gawryszewska, P. How minor structural changes generate major consequences in photophysical properties of RE coordination compounds; resonance effect, LMCT state, *J. Rare Earths* 2020, 38, 552.
[18] Judd, B. R. *Operator Techniques in Atomic Spectroscopy*, McGraw-Hill Book Company: New York, 1998.
[19] Beltrame, A. C. F.; Bispo Jr., A. G.; Canisares, F. S. M.; Fernandes, R. V.; Laureto, E.; Lima, S. A. M.; Pires, A. M. PMMA or PVDF films blended with β-diketonate tetrakis Eu^{III} or Tb^{III} complexes used as downshifting coatings of near-UV LEDs, *Soft Matter* 2023, 19, 3992 – 4000.
[20] Crist, B.; Schultz, J. M. Polymer spherulites: A critical review, *Prog. Polym. Sci.* 2016, 56, 1 – 63.
